# Existence of solutions to a diffusive shallow medium equation

**DOI:** 10.1007/s00028-020-00604-y

**Published:** 2020-07-25

**Authors:** Verena Bögelein, Nicolas Dietrich, Matias Vestberg

**Affiliations:** 1 Fachbereich Mathematik, Universität Salzburg, Hellbrunner Str. 34, 5020 Salzburg, Austria; 2 Department of Mathematics and Systems Analysis, Aalto University, P.O. Box 11100, 00076 Espoo, Finland

**Keywords:** Existence, Shallow medium equation, Doubly nonlinear parabolic equations, 35D30, 35K20, 35K65

## Abstract

In this article, we establish the existence of weak solutions to the shallow medium equation. We proceed by an approximation argument. First, we truncate the coefficients of the equation from above and below. Then, we prove convergence of the solutions of the truncated problem to a solution to the original equation.

## Introduction

In this work, we prove the existence of weak solutions to the Cauchy–Dirichlet problem associated with the doubly nonlinear equation

1.1 $$\begin{aligned} \partial _t u - \nabla \cdot \big ((u-z)^\alpha |\nabla u|^{p-2}\nabla u\big ) = f \quad \text { in } \Omega _T:=\Omega \times (0,T), \end{aligned}$$

where $\Omega \subset \mathbb {R}^n$ is an open bounded set, $z:\Omega \rightarrow \mathbb {R}$ and $f:\Omega _T\rightarrow \mathbb {R}$ are given sufficiently regular functions, and the parameters $\alpha$ and *p* are restricted to the range

$$\begin{aligned} p>1, \qquad \text { and }\qquad \alpha > 0. \end{aligned}$$

The solution *u* is required to satisfy $u\ge z$ so that the factor $(u-z)^\alpha$ is well defined. The term doubly nonlinear refers to the fact that the diffusion part depends nonlinearly both on the gradient and on the solution itself. In this article, we consider all $n\ge 2$, although the equation is typically used in models with only two spatial dimensions.

In the case $z=0$, (1.1) reduces to a standard doubly nonlinear equation which was studied already in [27] and [21], although using a different notion of weak solution. If we furthermore set $\alpha =0$, we recover the parabolic *p*-Laplace equation, whereas taking $p=2$ we obtain the porous medium equation, after a formal application of the chain rule. Thus, (1.1) can be seen as a generalization of many previously studied equations.

Local boundedness of weak solutions to (1.1) has been proved in [24] for sufficiently regular *f* and *z* in the slow diffusion case $\alpha +p >2$. The article focused on the case $p<2$, but the value of *p* does not really play a role in the arguments. In [25], local Hölder continuity was established for $p>2$. Existence of solutions to a Cauchy–Dirichlet problem corresponding to (1.1) was proved in the case of trivial topography $z=0$ and slow diffusion $\alpha + p >2$ in [3]. See also [4, 7, 15, 26] which study the existence of solutions to the Cauchy–Dirichlet problem for doubly nonlinear equations with general structure conditions. Existence, comparison properties and asymptotic behavior of solutions to a nonlinear parabolic problem related to our case have been studied in [8].

In the range $p > 2$, Eq. (1.1) is used to describe the dynamics of glaciers in the so-called shallow ice approximation; see for example Chapter 2 of [20] and the classic work [19]. Laboratory experiments, theoretical considerations and field measurements suggest that in these applications $p\approx 4$ provides the best description. This corresponds to the value 3 for the exponent appearing in the flow law for polycrystalline ice which was established in [13]. In the range $p<2$, Eq. (1.1) is used to model shallow water dynamics in situations such as floods and dam breaks; see [3, 11, 14]. Due to the variety of applications, we follow the terminology from [25] and use the term diffusive shallow medium equation or more concisely, DSM equation, to describe Eq. (1.1) for all $p>1$.

The given function *z* describes the elevation of the land on top of which the water or ice is moving, measured with respect to some arbitrarily chosen ground level. The value of *u* represents the height of the medium measured with respect to the same level. This provides a physical motivation for the condition $u\ge z$ which was imposed earlier. Even though *u* and *z* depend on the ground level, their difference $v:=u-z$ is invariant. Therefore, it is natural to reformulate the equation in terms of *v*; see (2.3) and Definition 2.1 of Sect. 2. As has been established in [24, 25], this formulation is also mathematically convenient.

The right-hand side *f* is a source term which can account for snowfall in the case of glaciers, and rainfall, evaporation or infiltration in the shallow water setting. Thus, its sign may vary in applications. However, in this paper we restrict ourselves to nonnegative source terms, which is necessary for the comparison principle argument utilized in Lemma 5.3. In physical applications, it is reasonable to assume that *f* is bounded, but our existence result requires only that the source term has sufficiently high integrability; see Sect. 2 for details.

The function *z* is required to belong to a first-order Sobolev space with sufficiently high exponent; see (2.1). In fact, the exponent is higher than the dimension of $\Omega$, which means that *z* is locally Hölder continuous. The space of admissible *z* contains for example all Lipschitz functions, which include the topographies occurring in realistic diffusion models.

Although the shallow medium equation has been studied in the literature, to our knowledge existence of weak solutions has not been established except for the trivial topography $z=0$. Our aim in this article is to close this gap. Our main result, Theorem 2.2, ensures the existence of weak solutions to the Cauchy–Dirichlet problem of the shallow medium equation (1.1).

## Setting and main result

In the following, we explain the precise setting and present the natural definition of weak solutions which was introduced in [24]. For the right-hand side $f :\Omega _T \rightarrow \mathbb {R}_{\ge 0}$ and the known function $z:\Omega \rightarrow \mathbb {R}$ and the initial datum $\psi :\Omega \rightarrow \mathbb {R}$, we assume

2.1 $$\begin{aligned} f\in L^{\sigma p'}(\Omega _T;\mathbb {R}_{\ge 0}), \quad z\in W^{1,\sigma \beta p}(\Omega ), \quad \Psi :=\psi -z \in L^\infty (\Omega ;\mathbb {R}_{\ge 0}), \end{aligned}$$

for some $\sigma >\frac{n+p}{p}$ and where $\beta$ is given by (2.4). Here, $p'=\frac{p}{p-1}$ denotes the Hölder conjugate of *p*. We consider the Cauchy–Dirichlet problem

2.2 $$\begin{aligned} \left\{ \begin{array}{ll} \partial _t u - \nabla \cdot \big ((u-z)^\alpha |\nabla u|^{p-2}\nabla u\big ) = f &{}\quad \text { in } \Omega _T,\\ u=z &{} \quad \text { on } \partial \Omega \times (0,T),\\ u(\cdot ,0)=\psi &{} \quad \text { in } \overline{\Omega }, \end{array} \right. \end{aligned}$$

for $u:\Omega _T\rightarrow \mathbb {R}$ with $u\ge z$. In order to motivate the natural definition of weak solutions, we reformulate (2.2)$_1$ in terms of $v:=u-z$. Formally applying the chain rule as in [24], we can write Eq. (2.2)$_1$ in the form

2.3 $$\begin{aligned} \partial _t v-\nabla \cdot \big (\beta ^{1-p}|\nabla v^\beta +\beta v^{\beta -1}\nabla z|^{p-2}(\nabla v^\beta +\beta v^{\beta -1}\nabla z)\big )= f, \end{aligned}$$

where

2.4 $$\begin{aligned} \beta :=1 + \frac{\alpha }{p-1}>1. \end{aligned}$$

Throughout the article, we will focus on this form of the equation. In order to simplify the notation, we denote the vector field appearing in the diffusion part of (2.3) as

2.5 $$\begin{aligned} A(v,\xi ):= \beta ^{1-p}|\xi +\beta v^{\beta -1}\nabla z|^{p-2}(\xi +\beta v^{\beta -1}\nabla z), \end{aligned}$$

for $v\ge 0$ and $\xi \in \mathbb {R}^n$, so that the Cauchy–Dirichlet problem (2.2) can be rewritten as

2.6 $$\begin{aligned} \left\{ \begin{array}{ll} \partial _t v-\nabla \cdot A(v,\nabla v^\beta )= f &{}\quad \text { in } \Omega _T,\\ v=0 &{} \quad \text { on } \partial \Omega \times (0,T),\\ v(\cdot ,0)=\psi -z &{} \quad \text { in } \overline{\Omega }, \end{array} \right. \end{aligned}$$

We arrive at the following definition of weak solutions by multiplying (2.3) by a smooth test function and integrating formally by parts.

### Definition 2.1

Suppose that *f*, *z* and $\psi$ satisfy (2.1). A function $u:\Omega _T\rightarrow \mathbb {R}$ is a weak solution to Eq. (1.1) if and only if $v:=u-z$ is nonnegative, $v^\beta \in L^p(0,T;W^{1,p}(\Omega ))$ and

2.7 $$\begin{aligned}&\iint _{\Omega _T} \big [A(v,\nabla v^\beta )\cdot \nabla \varphi - v\partial _t \varphi \big ]\mathrm {d}x\mathrm {d}t=\iint _{\Omega _T} f\varphi \mathrm {d}x\mathrm {d}t, \end{aligned}$$

for all $\varphi \in C^\infty _0(\Omega _T)$. Moreover, in this case we say that *v* is a solution to (2.3). If in addition $v\in C([0,T];L^{\beta +1}(\Omega ))$, $v^\beta \in L^p(0,T;W^{1,p}_0(\Omega ))$ and if $v(0)=\psi -z$, we say that *v* is a solution to the Cauchy–Dirichlet problem (2.6) and *u* is a solution to the Cauchy–Dirichlet problem (2.2).

Now, we can state our main existence theorem.

### Theorem 2.2

Suppose that *f*, *z* and $\psi$ satisfy (2.1). Then, there exists a weak solution to the Cauchy–Dirichlet problem (2.2) in the sense of Definition 2.1.

The main difficulty in the proof of Theorem 2.2 stems from the degeneracy of the shallow medium equation with respect to the solution and from the topography *z* which in a certain sense plays the role of an obstacle. For the construction of our solution, we proceed by an approximation argument. More precisely, we truncate the vector field *A* with respect to *v* from above and from below. For $k>1$, we let

$$\begin{aligned} A_k(v,\xi ):= A\big (T_k(v),\beta T_k(v)^{\beta -1}\xi \big ) \end{aligned}$$

for $v\in \mathbb {R}$ and $\xi \in \mathbb {R}^n$, where the truncation $T_k:\mathbb {R}\rightarrow \mathbb {R}$ is given by

$$\begin{aligned} T_k(s):= \min \big \{ k, \max \{ s, \tfrac{1}{k}\}\big \}. \end{aligned}$$

Then, we consider approximating solutions $v_k$ to a Cauchy–Dirichlet problem associated with the vector fields $A_k$ and with initial and lateral boundary data $\frac{1}{k}$, respectively, $\frac{1}{k}+\Psi$. The next crucial step is to investigate the properties of the approximating solutions. We prove the lower bound $v_k\ge \frac{1}{k}$ and an upper bound for $v_k$ independent of *k*. For large *k*, this allows us to conclude that

$$\begin{aligned} A_k(v_k,\nabla v_k)=A\big (v_k,\nabla v_k^\beta \big ). \end{aligned}$$

Next, we prove a uniform bound for the $L^p(\Omega _T)$-norms of the gradients $\nabla v_k^\beta$. This shows that there exists a weakly convergent subsequence of $v_k^\beta$ in $L^p(0,T;W^{1,p}(\Omega ))$ to some function *w*. By a delicate compactness argument (see Sect. 4), we identify the pointwise limit of $v_k$ as $v:=w^{\frac{1}{\beta }}$. In the next step, we use the differential equations for $v_k$, the ellipticity of the vector field *A* and the previously proved convergences to conclude pointwise convergence of the gradients $\nabla v_{k}^\beta \rightarrow \nabla v^\beta$ for a subsequence. This finally allows us to pass to the limit in the differential equations for $v_k$ and in turn to conclude that $u=v+z$ is the desired weak solution to the shallow medium equation.

We remark that there are also other ways of utilizing compactness to obtain the existence. For example, one could apply Corollary 7 of [23] to the sequence $(v_k^{\beta + 1})$, similarly as in the proof of Corollary 6.7 in [18]. One then needs to show that $\partial _t v_k^{\beta +1}$ exists and is bounded in a suitable space, but this can be done by using the mollified formulation (5.9) with a test function involving $([v_k]_h)^\beta$. The fact that $v_k$ itself has a gradient makes the argument possible. While such an approach is rather elegant, the compactness results that we obtain have the advantage of being directly applicable to similar problems in future research.

## Preliminaries

Here, we introduce some notation and present auxiliary tools that will be useful in the course of the paper.

### Notation

With $B_\varrho (y_o)$, we denote the open ball in $\mathbb {R}^m$, $m\in \mathbb {N}$ with radius $\varrho$ at center $y_o\in \mathbb {R}^m$. For $v,w \ge 0$, we define

3.1 $$\begin{aligned} \mathfrak {b}[v,w]&:= \tfrac{1}{\beta +1} (v^{\beta +1}-w^{\beta +1})- w^\beta (v-w) \nonumber \\&\;= \tfrac{\beta }{\beta +1} (w^{\beta +1}-v^{\beta +1})- v (w^\beta -v^\beta ), \end{aligned}$$

where $\beta$ is introduced in (2.4). For convenience, we will sometimes use the shorthand notation $w(t)=w(\cdot ,t)$ for $t\in [0,T]$. By *c*, we denote a generic constant which may change from line to line.

### Mollifications in time

Since weak solutions are not necessarily weakly differentiable with respect to time, we will use some mollification techniques. We first recall the definition of Steklov averages. Given a function $v\in L^1(\Omega _T)$ and $h\in (0,T)$, we define its Steklov mean by

3.2 $$\begin{aligned}{}[v]_h(x,t) := \frac{1}{h} \int _t^{t+h} v(x,s)\mathrm {d}s, \quad (x,t)\in \Omega \times (0,T-h), \end{aligned}$$

and the reversed Steklov mean by

3.3 $$\begin{aligned}{}[v]_{\bar{h}}(x,t) := \frac{1}{h} \int _{t-h}^{t} v(x,s)\mathrm {d}s \quad (x,t) \in \Omega \times (h,T). \end{aligned}$$

For some basic properties of Steklov averages, we refer to Chapter 1.3 of [9] and Sect. 9 of [10]. For porous medium-type equations and more generally doubly nonlinear equations, it is often necessary to work with the following so-called exponential time mollification, cf. [17]. For a function $v\in L^1(\Omega _T)$ and $h\in (0,T)$, we set

3.4 $$\begin{aligned} \llbracket v\rrbracket _h(x,t):=\frac{1}{h}\int ^t_0 e^\frac{s-t}{h}v(x,s)\mathrm {d}s. \end{aligned}$$

for $x\in \Omega$ and $t\in [0,T]$. Moreover, we define the reversed analogue by

$$\begin{aligned} \llbracket v\rrbracket _{\bar{h}}(x,t) :=\frac{1}{h}\int ^T_t e^\frac{t-s}{h}v(x,s)\mathrm {d}s. \end{aligned}$$

For details regarding the properties of the exponential mollification, we refer to [17, Lemma 2.2], [6, Lemma 2.2], and [26, Lemma 2.9]. The properties of the mollification that we will use have been collected for convenience into the following lemma:

#### Lemma 3.1

Suppose that $v \in L^1(\Omega _T)$, and let $p\in [1,\infty )$. Then, the mollification $\llbracket v\rrbracket _h$ defined in (3.4) has the following properties:

(i) If $$v\in L^p(\Omega _T)$$, then $$\llbracket v\rrbracket _h\in L^p(\Omega _T)$$,
  $$\begin{aligned} \Vert \llbracket v\rrbracket _h\Vert _{L^p(\Omega _T)}\le \Vert v\Vert _{L^p(\Omega _T)}, \end{aligned}$$
  and $\llbracket v\rrbracket _h\rightarrow v$ in $L^p(\Omega _T)$. A similar statement holds for $\llbracket v\rrbracket _{\bar{h}}$.
(ii) If $v\in L^p(\Omega _T)$, then $\llbracket v\rrbracket _h$ and $\llbracket v\rrbracket _{\bar{h}}$ have weak time derivatives belonging to $L^p(\Omega _T)$ given by
  $$\begin{aligned} \partial _t \llbracket v\rrbracket _h=\tfrac{1}{h}(v-\llbracket v\rrbracket _h),\quad \partial _t \llbracket v\rrbracket _{\bar{h}}=\tfrac{1}{h}(\llbracket v\rrbracket _{\bar{h}}-v). \end{aligned}$$
(iii) If $v\in L^p(0,T;W^{1,p}(\Omega ))$, then $\llbracket v\rrbracket _h\in L^p(0,T;W^{1,p}(\Omega ))$ and $\llbracket v\rrbracket _h\rightarrow v$ in
  $L^p(0,T;W^{1,p}(\Omega ))$ as $h\rightarrow 0$. Moreover, if $v\in L^p(0,T;W_0^{1,p}(\Omega ))$, then also
  $\llbracket v\rrbracket _h\in L^p(0,T;W_0^{1,p}(\Omega ))$. Similar statements hold for $\llbracket v\rrbracket _{\bar{h}}$.
(iv) If $v\in L^p(0,T;L^{p}(\Omega ))$, then $\llbracket v\rrbracket _h,\,\llbracket v\rrbracket _{\bar{h}} \in C([0,T];L^{p}(\Omega ))$.

### Elementary inequalities

We now recall some elementary inequalities that will be used later, and start by some useful estimates for the quantity $\mathfrak {b}[v,w]$ that was defined in (3.1). The proof can be found in [5, Lemma 2.2 and Lemma 2.3].

#### Lemma 3.2

Let $v,w \ge 0$ and $\beta > 1$. Then, there exists a constant $c\ge 1$ depending only on $\beta$ such that:

(i) $$\tfrac{1}{c}\big | w^{\frac{\beta +1}{2}}-v^{\frac{\beta +1}{2}} \big |^2 \le \mathfrak {b}[v,w] \le c \big | w^{\frac{\beta +1}{2}}-v^{\frac{\beta +1}{2}} \big |^2$$,
(ii) $$\mathfrak {b}[v,w] \ge \tfrac{1}{c} |v-w|^{\beta +1}$$,
(iii) $$\mathfrak {b}[v,w] \le c |v^{\beta }-w^{\beta }|^{\frac{\beta +1}{\beta }}$$.

The following algebraic lemma is standard and can be obtained by reasoning as in the proof of Lemma 5.1 in Chapter IX of [9].

#### Lemma 3.3

Let $p>1$. Then, for any $\xi ,\eta \in \mathbb {R}^n$ which in the case $p\in (1,2)$ are not both zero there holds

$$\begin{aligned} \big (|\xi |^{p-2}\xi - |\eta |^{p-2}\eta \big )\cdot (\xi -\eta ) \ge c\big (|\xi |^2 + |\eta |^2\big )^{\frac{p-2}{2}}|\xi -\eta |^2, \end{aligned}$$

where *c* is a constant depending only on *p*.

#### Remark 3.4

Let $p>1$ and $\xi ,\eta ,\zeta \in \mathbb {R}^n$ with either $\xi \not =\zeta$ or $\eta \not =\zeta$ in the case $p\in (1,2)$. Then, by Lemma 3.3 there holds

$$\begin{aligned}&\big (|\xi +\zeta |^{p-2}(\xi +\zeta ) - |\eta +\zeta |^{p-2}(\eta +\zeta )\big ) \cdot (\xi -\eta ) \\&\qquad = \big (|\xi +\zeta |^{p-2}(\xi +\zeta ) - |\eta +\zeta |^{p-2}(\eta +\zeta )\big ) \cdot \big ((\xi +\zeta )-(\eta +\zeta )\big ) \\&\qquad \ge \tfrac{1}{c} \big (|\xi +\zeta |^2 + |\eta +\zeta |^2\big )^{\frac{p-2}{2}}|\xi -\eta |^2 \\&\qquad \ge \tfrac{1}{c} \big (|\xi +\zeta | + |\eta +\zeta |\big )^{p-2}|\xi -\eta |^2. \end{aligned}$$

If $p\ge 2$, we use $|\xi -\eta |^2\le 2(|\xi +\zeta |^2+|\eta +\zeta |^2)$ to conclude

$$\begin{aligned} \big (|\xi +\zeta |^{p-2}(\xi +\zeta ) - |\eta +\zeta |^{p-2}(\eta +\zeta )\big ) \cdot (\xi -\eta ) \ge c\,|\xi -\eta |^p. \end{aligned}$$

#### Lemma 3.5

Let $p>1$. Then, for any $\xi ,\eta \in \mathbb {R}^n$ there holds

$$\begin{aligned} |\xi +\eta |^{p-2}(\xi +\eta )\cdot \xi \ge 2^{-p}|\xi |^p - 2^p|\eta |^p \end{aligned}$$

#### Proof

By Young’s inequality, we obtain

$$\begin{aligned} |\xi +\eta |^{p-2}(\xi +\eta )\cdot \xi&= |\xi +\eta |^{p} - |\xi +\eta |^{p-2}(\xi +\eta )\cdot \eta \\&\ge \tfrac{1}{2}|\xi +\eta |^{p} - 2^{p-1}|\eta |^p \\&\ge 2^{-p}|\xi |^p - 2^p|\eta |^p. \end{aligned}$$

This is the claimed inequality. $\square$

#### Lemma 3.6

Let $p>1$ and $\xi ,\eta \in \mathbb {R}^n$. In the case $p<2$, we also assume that either $\xi$ or $\eta$ is nonzero. Then, there exists a constant *c* depending only on *p* such that:

$$\begin{aligned} \big ||\xi |^{p-2}\xi -|\eta |^{p-2}\eta \big |\le c (|\eta |+ |\xi -\eta |)^{p-2}|\xi -\eta |. \end{aligned}$$

#### Proof

Applying [1, Lemma 2.2] with $\mu =0$, we obtain

$$\begin{aligned} \big ||\xi |^{p-2}\xi -|\eta |^{p-2}\eta \big |&\le c \big (|\eta |^2+ |\xi |^2\big )^{\frac{p-2}{2}}|\xi -\eta |\\&\le c (|\eta |+ |\xi |)^{p-2}|\xi -\eta |\\&\le 2^{|p-2|}c (|\eta |+ |\xi -\eta |)^{p-2}|\xi -\eta |, \end{aligned}$$

which is the desired estimate. $\square$

### Auxiliary tools

The following lemma can be proven using an inductive argument; see for example [9, § I, Lemma 4.1].

#### Lemma 3.7

Let $(Y_j)^\infty _{j=0}$ be a sequence of positive real numbers such that

$$\begin{aligned} Y_{j+1}\le C b^j Y^{1+\delta }_j, \end{aligned}$$

where $C, b >1$ and $\delta >0$. If

$$\begin{aligned} Y_0\le C^{-\frac{1}{\delta }}b^{-\frac{1}{\delta ^2}}, \end{aligned}$$

then $(Y_j)$ converges to zero as $j\rightarrow \infty$.

Next, we recall a well-known parabolic Sobolev inequality, which can be found for example in [9].

#### Lemma 3.8

Let $\Omega \subset \mathbb {R}^{n}$, $T>0$ and $\theta >0$. Suppose that $q>0$, $p>1$. Then, for every

$$\begin{aligned} u\in L^\infty \big (0,T;L^q(\Omega )\big ) \cap L^p\big (0,T;W^{1,p}_0(\Omega )\big ) \end{aligned}$$

we have

$$\begin{aligned} \iint _{\Omega _T} |u|^{p(1+\frac{q}{n})} \mathrm {d}x\mathrm {d}t&\le c\bigg [\mathop {{{\,\mathrm{ess\,sup}\,}}}\limits _{t\in (0,T)} \int _{\Omega \times \{t\}} |u|^q \mathrm {d}x\bigg ]^{\frac{p}{n}} \iint _{\Omega _T} |\nabla u|^p \mathrm {d}x\mathrm {d}t \end{aligned}$$

for a constant $$c=c(n,p,q,\Omega )$$.

We now present a version of the Cauchy–Peano existence theorem which will be used in connection with Galerkin’s method.

#### Lemma 3.9

Let *A* be an invertible $m\times m$ matrix, let $g:\mathbb {R}^m\rightarrow \mathbb {R}^m$ be continuous, and let $\tilde{f}:[0,T]\rightarrow \mathbb {R}^m$ be integrable. Let $y_o\in \mathbb {R}^m$. Then, there exists a $\delta >0$ and an absolutely continuous function $y:[0,\delta ]\rightarrow \mathbb {R}^m$ such that

$$\begin{aligned} A y'(t)&=g(y(t))+\tilde{f}(t), \quad \text {a.e. in }[0,\delta ], \\ y(0)&=y_o. \end{aligned}$$

#### Proof

Since the differential equation is equivalent to

$$\begin{aligned} y'(t)= A^{-1}\circ g(y(t))+ A^{-1}\circ \tilde{f}(t), \end{aligned}$$

and since $A^{-1}\circ g$ is continuous and $A^{-1}\circ \tilde{f}$ is integrable, we may assume $A=I_{m\times m}$. Fix $r>0$ and set $M:=\max _{\bar{B}_r(y_o)}|g|$. Define $\delta _1:=\tfrac{r}{2M+1}$ and pick $\delta _2>0$ so small that

$$\begin{aligned} \int ^{\delta _2}_0 |\tilde{f}| \mathrm {d}t < \frac{r}{2}. \end{aligned}$$

We choose $\delta =\min \{\delta _1,\delta _2\}$. Denote by $c(y_o)$ the constant function $[0,\delta ]\rightarrow \mathbb {R}^m$ taking the value $y_o$ for all $t\in [0,\delta ]$, and define $\widetilde{F}(t):=M+|\tilde{f}(t)|$. Consider the subset

$$\begin{aligned} K:=\bigg \{u\in \bar{B}_r(c(y_o))\,:\, |u(t_1)-u(t_2)|\le \int ^{t_2}_{t_1} \widetilde{F}(t)\mathrm {d}t, \quad 0\le t_1\le t_2 \le \delta \bigg \} \end{aligned}$$

of the vector space $C([0,\delta ];\mathbb {R}^m)$ equipped with the sup-norm. The set *K* is convex and closed. Furthermore, the elements of *K* are equicontinuous pointwise bounded functions, so the Arzelà–Ascoli theorem guarantees that *K* is compact. We note that we have a well-defined map $T:K\rightarrow K$ defined by

$$\begin{aligned} (T u)(t)= y_o+\int ^t_0 \big [g(u(s))+ \tilde{f}(s)\big ]\mathrm {d}s. \end{aligned}$$

Since

$$\begin{aligned} |Tu(t)-Tv(t)|\le \int ^t_0 |g(u(s))-g(v(s))|\mathrm {d}s \le \delta \sup _{s\in [0,\delta ]}|g(u(s))-g(v(s))|, \end{aligned}$$

the uniform continuity of *g* on the compact set $\bar{B}_r(y_o)$ shows that *T* is continuous $K\rightarrow K$. Thus, Schauder’s fixed point theorem guarantees that there exists at least one $y\in K$ such that $Ty=y$. But then *y* is a solution to the original problem. $\square$

### Time continuity

We have taken a certain time continuity as part of the definition of a solution to the Cauchy–Dirichlet problem. Our next goal is to show that all weak solutions vanishing on the lateral boundary and having sufficiently high integrability automatically have this type of time continuity. The extra integrability condition $v\in L^{\beta +1}(\Omega _T)$ occurring in the two following lemmas is redundant in the case $\alpha +p\ge 2$, since then $\beta p\ge \beta + 1$. Note that we will only apply the results to the bounded solution found in Sect. 6, which obviously satisfies the extra integrability condition.

#### Lemma 3.10

For all $v\in L^{\beta +1}(\Omega _T;\mathbb {R}_{\ge 0})$ with $v^\beta \in L^p(0,T;W^{1,p}_0(\Omega ))$ satisfying (2.7), all $\zeta \in W^{1,\infty }([0,T];\mathbb {R}_{\ge 0})$ satisfying $\zeta (0)=\zeta (T)=0$, and all *w* in

$$\begin{aligned} \mathcal {V}:=\big \{ w \in L^{\beta +1}(\Omega _T)\,|\, w^\beta \in L^p(0,T;W^{1,p}_0(\Omega )), \partial _t w^\beta \in L^{\frac{\beta +1}{\beta }}(\Omega _T) \big \}, \end{aligned}$$

we have

3.5 $$\begin{aligned} \iint _{\Omega _T}&\zeta ' \mathfrak {b}[v,w]\mathrm {d}x \mathrm {d}t\nonumber \\&= \iint _{\Omega _T} \zeta \Big [\partial _t w^\beta (v-w) + A(v,\nabla v^\beta )\cdot (\nabla v^\beta -\nabla w^\beta ) - f (v^\beta -w^\beta )\Big ] \mathrm {d}x\mathrm {d}t. \end{aligned}$$

#### Proof

We use (2.7) with the test function $\varphi _h=\zeta (w^\beta -\llbracket v^\beta \rrbracket _h)$. This is possible since we can find $\varphi _{h,j}\in C^\infty _0(\Omega _T)$ such that $\varphi _{h,j}\rightarrow \varphi _h$ in $L^p(0,T;W^{1,p}(\Omega ))$ and $\partial _t \varphi _{h,j} \rightarrow \partial _t \varphi _h$ in $L^\frac{\beta +1}{\beta }(\Omega _T)$. We see immediately that

3.6 $$\begin{aligned} \lim _{h\downarrow 0} \iint _{\Omega _T}A(v,\nabla v^\beta ) \cdot \nabla \varphi _h \mathrm {d}x \mathrm {d}t = \iint _{\Omega _T}A(v,\nabla v^\beta ) \cdot (\nabla w^\beta -\nabla v^\beta )\zeta \mathrm {d}x \mathrm {d}t, \end{aligned}$$

and

3.7 $$\begin{aligned} \lim _{h\downarrow 0} \iint _{\Omega _T}f \varphi _h \mathrm {d}x \mathrm {d}t = \iint _{\Omega _T} f \zeta (w^\beta -v^\beta )\mathrm {d}x\mathrm {d}t. \end{aligned}$$

The parabolic term is treated as follows:

$$\begin{aligned} \iint _{\Omega _T}&v\partial _t \varphi _h \mathrm {d}x\mathrm {d}t\\&= \iint _{\Omega _T} \zeta ' v (w^\beta -\llbracket v^\beta \rrbracket _h) + \zeta v(\partial _t w^\beta -\partial _t \llbracket v^\beta \rrbracket _h)\mathrm {d}x\mathrm {d}t\\&= \iint _{\Omega _T} \zeta v\partial _t w^\beta - \zeta \llbracket v^\beta \rrbracket _h^\frac{1}{\beta }\partial _t \llbracket v^\beta \rrbracket _h + \zeta ' v (w^\beta -\llbracket v^\beta \rrbracket _h) \\&\quad + \zeta (\llbracket v^\beta \rrbracket _h^\frac{1}{\beta }-v)\partial _t \llbracket v^\beta \rrbracket _h\mathrm {d}x\mathrm {d}t\\&\le \iint _{\Omega _T} \zeta v \partial _t w^\beta + \zeta ' \tfrac{\beta }{\beta +1}\llbracket v^\beta \rrbracket _h^\frac{\beta +1}{\beta } + \zeta ' v (w^\beta -\llbracket v^\beta \rrbracket _h) \mathrm {d}x\mathrm {d}t. \end{aligned}$$

In the third step, we have used Lemma 3.1 (ii) to conclude that the last term is nonpositive, and the second term has been integrated by parts. The limit of the parabolic term as $h\rightarrow 0$ exists due to (3.6), (3.7) and (2.7) and satisfies the estimate

3.8 $$\begin{aligned}&\lim _{h\downarrow 0} \iint _{\Omega _T} v\partial _t \varphi _h \mathrm {d}x\mathrm {d}t \le \iint _{\Omega _T} \zeta v \partial _t w^\beta + \zeta ' \big (\tfrac{\beta }{\beta +1}v^{\beta +1} + v (w^\beta -v^\beta ) \big ) \mathrm {d}x\mathrm {d}t\nonumber \\&\qquad = \iint _{\Omega _T} \zeta (v-w) \partial _t w^\beta + \zeta \tfrac{\beta }{\beta +1}\partial _t (w^\beta )^\frac{\beta +1}{\beta } + \zeta ' \big (\tfrac{\beta }{\beta +1}v^{\beta +1} + v (w^\beta -v^\beta ) \big ) \mathrm {d}x\mathrm {d}t \nonumber \\&\qquad = \iint _{\Omega _T} \zeta (v-w) \partial _t w^\beta - \zeta ' \mathfrak {b}[v,w] \mathrm {d}x \mathrm {d}t. \end{aligned}$$

In the last step, we integrate the second term by parts. Combining (3.6), (3.7) and (3.8), we have verified “$\le$” in (3.5). The reverse inequality follows in a similar way by taking $\varphi =\zeta (w^\beta -\llbracket v^\beta \rrbracket _{\bar{h}})$ as a test function.$\square$

Using the previous lemma, we can now conclude the time continuity.

#### Lemma 3.11

Every $v\in L^{\beta +1}(\Omega _T;\mathbb {R}_{\ge 0})$ with $v^\beta \in L^p(0,T;W^{1,p}_0(\Omega ))$ satisfying (2.7) has a representative in $C([0,T];L^{\beta +1}(\Omega ))$.

#### Proof

Take $\psi \in C^\infty (\mathbb {R};[0,1])$ such that $\psi (t)=1$ for $t\le \tfrac{1}{2} T$, $\psi (t)=0$ for $t>\tfrac{3}{4} T$ and $|\psi '|\le \tfrac{8}{T}$. For $\tau \in (0, \tfrac{1}{2} T)$ and $\varepsilon >0$ so small that $\tau +\varepsilon < \tfrac{1}{2} T$, define

$$\begin{aligned} \chi ^\tau _\varepsilon (t)= {\left\{ \begin{array}{ll} 0, &{} t<\tau \\ \frac{1}{\varepsilon }(t-\tau ), &{} t\in [\tau , \tau +\varepsilon ] \\ 1, &{} t> \tau +\varepsilon . \end{array}\right. } \end{aligned}$$

Using identity (3.5) from Lemma 3.10 with $\zeta =\chi ^\tau _\varepsilon \psi$ and $w=(\llbracket v^\beta \rrbracket _{\bar{h}})^\frac{1}{\beta }$, we obtain

$$\begin{aligned}&\varepsilon ^{-1}\int ^{\tau +\varepsilon }_\tau \int _\Omega \mathfrak {b}[v,w]\mathrm {d}x \mathrm {d}t\\&\quad = \iint _{\Omega _T}\big [A(v,\nabla v^\beta )\cdot (\nabla v^\beta -\nabla w^\beta ) + (v-w)\partial _t w^\beta \big ]\zeta \mathrm {d}x \mathrm {d}t\\&\qquad +\iint _{\Omega _T} f (w^\beta -v^\beta )\zeta \mathrm {d}x\mathrm {d}t - \iint _{\Omega _T}\mathfrak {b}[v,w]\psi ' \mathrm {d}x \mathrm {d}t\\&\quad \le \iint _{\Omega _T}\big [|A(v,\nabla v^\beta )||\nabla v^\beta -\nabla \llbracket v^\beta \rrbracket _{\bar{h}} | + |f||\llbracket v^\beta \rrbracket _{\bar{h}}-v^\beta |\big ] \mathrm {d}x \mathrm {d}t\\&\qquad +\frac{c}{T}\iint _{\Omega _T} |v^\beta - \llbracket v^\beta \rrbracket _{\bar{h}} |^\frac{\beta +1}{\beta }\mathrm {d}x \mathrm {d}t. \end{aligned}$$

Here, we used Lemma 3.1 (ii) to conclude that the term involving $\partial _t w^\beta$ is nonpositive. Also, we have made use of Lemma 3.2 (iii) to estimate $\mathfrak {b}[v,w]$. Passing to the limit $\varepsilon \rightarrow 0$, we see that

$$\begin{aligned} \int _\Omega \mathfrak {b}[v,w](\cdot ,\tau ) \mathrm {d}x&\le \iint _{\Omega _T}\big [|A(v,\nabla v^\beta )||\nabla v^\beta -\nabla \llbracket v^\beta \rrbracket _{\bar{h}} | + |f||\llbracket v^\beta \rrbracket _{\bar{h}}-v^\beta |\big ] \mathrm {d}x \mathrm {d}t\\&\quad +\frac{c}{T}\iint _{\Omega _T} |v^\beta - \llbracket v^\beta \rrbracket _{\bar{h}} |^\frac{\beta +1}{\beta }\mathrm {d}x \mathrm {d}t \end{aligned}$$

for all $\tau \in (0,\tfrac{T}{2}){\setminus } N_h$ where $N_h$ is a set of measure zero. Using Lemma 3.2 (ii), we find a lower bound for the integrand on the left-hand side:

$$\begin{aligned} |v-w|^{\beta +1} \le c\, \mathfrak {b}[v,w]. \end{aligned}$$

Taking now a sequence $h_j\downarrow 0$ and setting $w_j:= ( [\![v^\beta ]\!]_{\bar{h}_j})^\frac{1}{\beta }$ and $N:=\cup N_{h_j}$, we see that

3.9 $$\begin{aligned} \lim _{j\rightarrow \infty } \sup _{t\in [0,\frac{T}{2}]{\setminus } N} \int _{\Omega } |v-w_j|^{\beta +1}(\cdot ,t) \mathrm {d}x = 0 \end{aligned}$$

Observe that every map of the form $w=(\llbracket v^\beta \rrbracket _{\bar{h}})^\frac{1}{\beta }$ is continuous $[0,T]\rightarrow L^{\beta +1}(\Omega )$ since

$$\begin{aligned}&|w(x,s)-w(x,t)|^{\beta +1}\le |w^\beta (x,s)-w^\beta (x,t)|^\frac{\beta +1}{\beta }\\&\quad =|\llbracket v^\beta \rrbracket _{\bar{h}}(x,s) - \llbracket v^\beta \rrbracket _{\bar{h}}(x,t)|^\frac{\beta +1}{\beta }, \end{aligned}$$

and $\llbracket v^\beta \rrbracket _{\bar{h}}$ is continuous $[0,T]\rightarrow L^\frac{\beta +1}{\beta }(\Omega )$ by Lemma 3.1 (iv). Because of the uniform limit (3.9), *v* has a representative which is continuous on $[0,\tfrac{T}{2}]{\setminus } N$ and since *N* has measure zero we find a representative which is continuous on $[0,\tfrac{T}{2}]$. The continuity on $[\tfrac{T}{2},T]$ follows from a similar argument, but taking $w=(\llbracket v^\beta \rrbracket _h)^\frac{1}{\beta }$ and reflecting $\chi ^\tau _\varepsilon$ and $\psi$ on the interval [0, *T*]. $\square$

## Compactness results

In this section, we provide some compactness results which could be of interest in their own. They are consequences of the well-known compactness results in [23]. Such results are necessary when dealing with porous medium-type equations and doubly nonlinear equations. For the purpose of this paper, we only need Corollary 4.6. Since there is not much extra effort to obtain other versions, we preferred to include them as well for possible future applications. Some results in this direction were previously obtained for uniformly bounded functions that are piecewise constant in time; see [16, Lemma 8] and the references therein.

For a family of functions $F \subset L^{1}(0,T;L^1(\Omega ;\mathbb {R}^N))$ with $N\ge 1$, we denote

$$\begin{aligned} F^m:=\big \{|f|^{m-1}f:f\in F\big \} \quad \text{ for } m>0. \end{aligned}$$

We start with an Arzéla–Ascoli-type theorem. It will be the basis for all the other compactness results. For a map $f:[0,T]\rightarrow X$, we define the translated function $\tau _h f:[0,T-h]\rightarrow X$ by $\tau _h f(t)=f(t+h)$. We can now formulate the following theorem [23, § 3, Theorem 1]:

### Theorem 4.1

Let $1\le p\le \infty$, $T>0$ and *B* be a Banach space. Let $F\subset L^p(0,T;B)$. Then, *F* is relatively compact in $L^p(0,T;B)$ for $1\le p<\infty$, or in *C*([0, *T*]; *B*) for $p=\infty$ if and only if

4.1 $$\begin{aligned} \bigg \{\int _{t_1}^{t_2} f(t) \,\mathrm {d}t: f\in F\bigg \} \quad \text{ is } \text{ relatively } \text{ compact } \text{ in } B, \quad \forall \,0<t_1<t_2<T \end{aligned}$$

and

4.2 $$\begin{aligned} \Vert \tau _h f - f\Vert _{L^p(0,T-h;B)}\rightarrow 0 \quad \text{ as } h\downarrow 0, \quad \text{ uniformly } \text{ for } f\in F. \end{aligned}$$

Using Theorem 4.1, we obtain the following result.

### Theorem 4.2

Let $m \in (0,\infty )$, $1\le p\le \infty$, $1\le q\le \infty$. Define $\theta :=\max \{1, m p\}$ and $\mu :=\max \{1,mq\}$. Let $T>0$ and let $\Omega \subset \mathbb {R}^n$ be a bounded domain. Let *X* be a Banach space with compact embedding $X\hookrightarrow L^q(\Omega ;\mathbb {R}^N)$. Moreover, let $F \subset L^{\theta }(0,T;L^\mu (\Omega ;\mathbb {R}^N))$ such that $F^m\subset L^1(0,T;X)$. We assume that

4.3 $$\begin{aligned} F^m \text{ is } \text{ bounded } \text{ in } L^{p}(0,T;L^q(\Omega ;\mathbb {R}^N)) \text{ if } m\ge 1 \end{aligned}$$

and

4.4 $$\begin{aligned} F^m \text{ is } \text{ bounded } \text{ in } L^1(0,T;X) \end{aligned}$$

and

4.5 $$\begin{aligned} \Vert \tau _h f - f\Vert _{L^\theta (0,T-h;L^\mu (\Omega ;\mathbb {R}^N))}\rightarrow 0 \quad \text{ as } h\downarrow 0, \quad \text{ uniformly } \text{ for } f\in F. \end{aligned}$$

Then, $F^m$ is relatively compact in $L^{p}(0,T;L^q(\Omega ;\mathbb {R}^N))$ for $1\le p<\infty$. For $p=\infty$, it is relatively compact in $C([0,T];L^q(\Omega ;\mathbb {R}^N))$.

Before proceeding with the proof, we note that the assumption $F \subset L^{\theta }(0,T;L^\mu (\Omega ;\mathbb {R}^N))$ guarantees that $F^m\subset L^p(0,T;L^q(\Omega ;\mathbb {R}^N))$. In the case $m\ge 1$, we additionally need to assume that $F^m$ is bounded in $L^p(0,T;L^q(\Omega ;\mathbb {R}^N))$. The assumption $F^m\subset L^1(0,T;X)$ is to be understood in the following sense: For every $f\in F$, there is an element of $L^1(0,T;X)$ whose composition with the inclusion $X\hookrightarrow L^q(\Omega )$ coincides with $f^m\in L^p(0,T;L^q(\Omega ;\mathbb {R}^N))$.

### Proof of Theorem 4.2

Our aim is to apply Theorem 4.1 to the functions $F^m$ with $B=L^q(\Omega ;\mathbb {R}^N)$. From (4.4), we know that $F^m$ is bounded in $L^1(t_1,t_2;X)$ for any $0<t_1<t_2<T$. Due to the compact embedding $X\subset L^q(\Omega ;\mathbb {R}^N)$, this implies that $\{\int _{t_1}^{t_2} f^m(t) \,\mathrm {d}t: f^m\in F^m\}$ is relatively compact in $L^q(\Omega ;\mathbb {R}^N)$ for any $0<t_1<t_2<T$. Hence, (4.1) is satisfied. Next, we verify assumption (4.2). Suppose first $p<\infty$. If $m\in (0,1)$, we have

$$\begin{aligned} \Vert \tau _h f^m - f^m\Vert ^p_{L^p(0,T-h;L^q(\Omega ;\mathbb {R}^N))}&= \int _0^{T-h} \bigg [\int _\Omega |f^m(t+h)-f^m(t)|^q \,\mathrm {d}x \bigg ]^{\frac{p}{q}} \mathrm {d}t\\&\le c\int _0^{T-h} \bigg [\int _\Omega |f(t+h)-f(t)|^{mq} \,\mathrm {d}x \bigg ]^{\frac{p}{q}} \mathrm {d}t\\&\le c\int _0^{T-h} \bigg [\int _\Omega |f(t+h)-f(t)|^{\mu } \,\mathrm {d}x \bigg ]^{\frac{m p}{\mu }} \mathrm {d}t \\&\le c\,\Vert \tau _h f - f\Vert ^{mp}_{L^\theta (0,T-h;L^\mu (\Omega ;\mathbb {R}^N))}. \end{aligned}$$

Otherwise, if $m\ge 1$, then $\theta =mp$ and $\mu =mq$ and we compute

$$\begin{aligned}&\Vert \tau _h f^m - f^m\Vert ^p_{L^p(0,T-h;L^q(\Omega ;\mathbb {R}^N))} = \int _0^{T-h} \bigg [\int _\Omega |f^m(t+h)-f^m(t)|^q \,\mathrm {d}x \bigg ]^{\frac{p}{q}} \mathrm {d}t\\&\qquad \le c\int _0^{T-h} \bigg [\int _\Omega |f(t+h)-f(t)|^q \big (|f(t+h)| + |f(t)|\big )^{(m-1)q} \,\mathrm {d}x \bigg ]^{\frac{p}{q}} \mathrm {d}t\\&\qquad \le c\int _0^{T-h} \bigg [\int _\Omega |f(t+h)-f(t)|^{\mu } \,\mathrm {d}x \bigg ]^{\frac{p}{\mu }} \\&\quad \qquad \times \bigg [\int _\Omega \big (|f(t+h)| + |f(t)|\big )^{mq} \,\mathrm {d}x \bigg ]^{\frac{(m-1)p}{mq}}\mathrm {d}t \\&\quad \qquad \le c\,\Vert \tau _h f - f\Vert ^{\frac{\theta }{m}}_{L^\theta (0,T-h;L^\mu (\Omega ;\mathbb {R}^N))} \Vert f^m\Vert ^{(1-\frac{1}{m})p}_{L^p(0,T-h;L^q(\Omega ;\mathbb {R}^N))}. \end{aligned}$$

This ensures that also (4.2) is satisfied. In the case $p=\infty$, one must again consider the two ranges for *m*. The calculations are similar. Therefore, Theorem 4.1 yields the compactness of $F^m$ in $L^{p}(0,T;L^q(\Omega ;\mathbb {R}^N))$ for $1\le p<\infty$, or in $C(0,T;L^q(\Omega ;\mathbb {R}^N))$ for $p=\infty$. $\square$

This interpolation lemma is from [23, § 8, Lemma 8].

### Lemma 4.3

Let *B*, *X*, *Y* be Banach spaces with $X\subset B\subset Y$ and compact embedding $X \hookrightarrow B$. Then, for any $\eta >0$ there exists $M_\eta >0$ such that

$$\begin{aligned} \Vert v\Vert _B \le \eta \Vert v\Vert _X + M_\eta \Vert v\Vert _Y \quad \text{ for } \text{ any } v\in X. \end{aligned}$$

Let *X* be a Banach space and let $q,\mu \in [1,\infty )$. Throughout the rest of this section, we will consider compact embeddings $T:X\rightarrow L^q(\Omega ;\mathbb {R}^N)$ and $S:L^{\mu '}(\Omega ;\mathbb {R}^N)\rightarrow X'$ that are compatible in the following way. For any $v\in X$ for which it happens that the function *Tv* belongs to $L^\mu (\Omega ;\mathbb {R}^N)$ and for any $w\in L^{\mu '}(\Omega ;\mathbb {R}^N)$, we have

4.6 $$\begin{aligned} \int _\Omega Tv \cdot w\mathrm {d}x = \langle v, S w\rangle , \end{aligned}$$

where $\langle \cdot ,\cdot \rangle$ denotes the dual pairing of *X* and $X'$.

We need the following more complicated version of Lemma 4.3.

### Lemma 4.4

Let $m\in (0,\infty )$, $p,q,\mu \in [1,\infty )$, $\theta :=\max \{1,m p\}$, $T>0$, and $\Omega \subset \mathbb {R}^n$ be a bounded domain and *X*, *Y* be Banach spaces such that $X\subset L^q(\Omega ;\mathbb {R}^N)$ and $L^{\mu '}(\Omega ;\mathbb {R}^N)\subset X'\subset Y$ with compact embeddings $T: X \hookrightarrow L^q(\Omega ;\mathbb {R}^N)$ and $S: L^{\mu '}(\Omega ;\mathbb {R}^N)\hookrightarrow X'$ satisfying (4.6). Then, for any $\eta >0$ there exists $M_\eta >0$ such that

$$\begin{aligned}&\Vert \tau _h f^m -f^m\Vert _{L^{p}(0,T-h;L^{q}(\Omega ;\mathbb {R}^N))} \\&\quad \le \Vert f^m\Vert _{L^p(0,T;X)}^{\frac{m}{m+1}} \bigg [ \eta \Big [\Vert f^m\Vert _{L^{p}(0,T;X)}^{\frac{1}{m+1}} + \Vert f\Vert _{L^{\theta }(0,T;L^{\mu '}(\Omega ;\mathbb {R}^N))}^{\frac{m}{m+1}} \Big ]\\&\qquad + M_{\eta } \Vert \tau _h f-f\Vert _{L^{\theta }(0,T-h;Y)}^{\frac{m}{m+1}} \bigg ]. \end{aligned}$$

for any $f\in L^{\theta }(0,T;L^{\mu '}(\Omega ;\mathbb {R}^N))$ with $f^m\in L^p(0,T;X)\cap L^p(0,T;L^\mu (\Omega ;\mathbb {R}^N))$.

Note that the assumption $f^m\in L^p(0,T;X)$ must be understood in the same sense as in Lemma 4.2. In the sequel, we do not explicitly write out the embeddings. It will be clear from the context when they should be present.

### Proof

We consider $v\in L^p(0,\tau ;X)\cap L^p(0,\tau ;L^\mu (\Omega ;\mathbb {R}^N))$ (i.e., the composition of *v* with the embedding *T* results in a function belonging to $L^p(0,\tau ;L^\mu (\Omega ;\mathbb {R}^N))$) and $w\in L^{\theta }(0,\tau ;L^{\mu '}(\Omega ;\mathbb {R}^N))$ with $\tau \in (0,T]$. Since $L^{\mu '}(\Omega ;\mathbb {R}^N)\subset X'$, we know that $\langle v(t),w(t)\rangle$ is defined for a.e. $t\in (0,\tau )$, where $\langle \cdot ,\cdot \rangle$ denotes the dual pairing of *X* and $X'$. Hence, we may calculate

4.7 $$\begin{aligned}&\bigg [\int _0^{\tau } \bigg |\int _\Omega v \cdot w \,\mathrm {d}x \bigg |^{\frac{\theta p}{\theta +p}} \mathrm {d}t \bigg ]^{\frac{1}{p}}\nonumber \\&\quad = \bigg [\int _0^{\tau } |\langle v, w\rangle |^{\frac{\theta p}{\theta +p}} \,\mathrm {d}t \bigg ]^{\frac{1}{p}} \nonumber \\&\quad \le \bigg [\int _0^{\tau } \Vert v\Vert _X^{\frac{\theta p}{\theta +p}} \Vert w\Vert _{X'}^{\frac{\theta p}{\theta +p}} \,\mathrm {d}t \bigg ]^{\frac{1}{p}} \nonumber \\&\quad \le \Vert v\Vert _{L^p(0,\tau ;X)}^{\frac{\theta }{\theta +p}} \Vert w\Vert _{L^\theta (0,\tau ;X')}^{\frac{\theta }{\theta +p}} , \end{aligned}$$

where in the last line we applied Hölder’s inequality. Here, we have omitted the embeddings *S* and *T* to simplify the notation. In particular, choosing $v=f^m$ and $w=f$ in (4.7) yields

4.8 $$\begin{aligned} \bigg [\int _0^{T} \bigg [\int _\Omega |f|^{m+1} \,\mathrm {d}x \bigg ]^{\frac{\theta p}{\theta +p}} \mathrm {d}t \bigg ]^{\frac{1}{p}}&\le \Vert f^m\Vert _{L^p(0,T;X)}^{\frac{\theta }{\theta +p}} \Vert f\Vert _{L^\theta (0,T;X')}^{\frac{\theta }{\theta +p}} \nonumber \\&\le c\, \Vert f^m\Vert _{L^p(0,T;X)}^{\frac{\theta }{\theta +p}} \Vert f\Vert _{L^\theta (0,T;L^{\mu }(\Omega ;\mathbb {R}^N))}^{\frac{\theta }{\theta +p}} . \end{aligned}$$

For $t\in [0,T-h]$, set $\Omega ^t=\Omega \cap \{\tau _h f^m(t)\ne f^m(t)\}$. Applying in turn Hölder’s inequality, Lemma 3.3 and (4.7) with the choice $v=\tau _h f^m-f^m$ and $w=\tau _h f-f$ leads to

$$\begin{aligned} \text{ I }&:= \bigg [\int _0^{T-h} \bigg |\int _{\Omega ^t} \big (|\tau _hf^m| + |f^m|\big )^{\frac{1-m}{m}} |\tau _hf^m-f^m|^{2} \,\mathrm {d}x\bigg |^{\frac{m p}{m+1}} \mathrm {d}t \bigg ]^{\frac{1}{p}} \nonumber \\&\le T^{\frac{\theta -m p}{p\theta (m+1)}} \bigg [\int _0^{T-h} \bigg |\int _{\Omega ^t} \big (|\tau _hf^m| + |f^m|\big )^{\frac{1-m}{m}} \big |\tau _hf^m-f^m\big |^{2} \,\mathrm {d}x\bigg |^{\frac{\theta p}{\theta +p}} \mathrm {d}t \bigg ]^{\frac{1}{p}\cdot \frac{m(\theta +p)}{\theta (m+1)}} \nonumber \\&\le c\bigg [\int _0^{T-h} \bigg |\int _\Omega \big (\tau _h f^m-f^m\big ) \cdot (\tau _h f-f) \,\mathrm {d}x \bigg |^{\frac{\theta p}{\theta +p}} \mathrm {d}t \bigg ]^{\frac{1}{p}\cdot \frac{m(\theta +p)}{\theta (m+1)}} \nonumber \\&\le c\,\Vert f^m\Vert _{L^p(0,T;X)}^{\frac{m}{m+1}} \Vert \tau _h f-f\Vert _{L^\theta (0,T-h;X')}^{\frac{m}{m+1}}, \end{aligned}$$

for some constant $c=c(m)\ge 1$. The set $\Omega ^t$ was introduced instead of $\Omega$ to avoid dividing by zero in the case $m>1$. Now, let $\eta _1>0$. By Lemma 4.3, there exists $M_{\eta _1}>0$ such that

$$\begin{aligned} \text{ I }&\le c\,\Vert f^m\Vert _{L^p(0,T;X)}^{\frac{m}{m+1}}\\&\qquad \times \bigg [ \eta _1^{\frac{m}{m+1}} \Vert \tau _h f-f\Vert _{L^{\theta }(0,T-h;L^{\mu '}(\Omega ;\mathbb {R}^N))}^{\frac{m}{m+1}} + M_{\eta _1}^{\frac{m}{m+1}} \Vert \tau _h f-f\Vert _{L^{\theta }(0,T-h;Y)}^{\frac{m}{m+1}} \bigg ] \\&\le c\,\Vert f^m\Vert _{L^p(0,T;X)}^{\frac{m}{m+1}} \bigg [ \eta _1^{\frac{m}{m+1}} \Vert f\Vert _{L^{\theta }(0,T;L^{\mu '}(\Omega ;\mathbb {R}^N))}^{\frac{m}{m+1}} + M_{\eta _1}^{\frac{m}{m+1}} \Vert \tau _h f-f\Vert _{L^{\theta }(0,T-h;Y)}^{\frac{m}{m+1}} \bigg ], \end{aligned}$$

where $c=c(m,p)$. If $m\le 1$, we therefore immediately conclude that

$$\begin{aligned} \Vert \tau _h f^m&-f^m\Vert _{L^{p}(0,T-h;L^{\frac{m+1}{m}}(\Omega ;\mathbb {R}^N))} \le c\, \text{ I }\\&\le c\, \Vert f^m\Vert _{L^p(0,T;X)}^{\frac{m}{m+1}} \bigg [ \eta _1^{\frac{m}{m+1}} \Vert f\Vert _{L^{\theta }(0,T;L^{\mu '}(\Omega ;\mathbb {R}^N))}^{\frac{m}{m+1}} + M_{\eta _1}^{\frac{m}{m+1}} \Vert \tau _h f-f\Vert _{L^{\theta }(0,T-h;Y)}^{\frac{m}{m+1}} \bigg ], \end{aligned}$$

where $c=c(m,p)$. If $m> 1$, we use Hölder’s inequality to estimate $\text{ I }$ from below and get with the abbreviation $F:=(|\tau _h f^m| + |f^m|)^{\frac{1}{m}}$, inequality (4.8) and Young’s inequality that

$$\begin{aligned}&\Vert \tau _h f^m -f^m\Vert _{L^{p}(0,T-h;L^{\frac{m+1}{m}}(\Omega ;\mathbb {R}^N))} \\&\quad = \bigg [\int _0^{T-h} \bigg [\int _{\Omega ^t} F^{\frac{(m+1)(m-1)}{2m}}F^{\frac{(m+1)(1-m)}{2m}} |\tau _h f^m-f^m|^{\frac{m+1}{m}} \,\mathrm {d}x\bigg ]^{\frac{m p}{m+1}} \mathrm {d}t \bigg ]^\frac{1}{p}\\&\quad \le \bigg [\int _0^{T-h} \bigg [\int _\Omega F^{m+1} \,\mathrm {d}x\bigg ]^{\frac{m p}{m+1}} \mathrm {d}t \bigg ]^{\frac{m-1}{2m p}} \bigg [\int _0^{T-h} \bigg [\int _{\Omega ^t} F^{1-m} |\tau _h f^m-f^m|^{2} \,\mathrm {d}x\bigg ]^{\frac{m p}{m+1}} \mathrm {d}t \bigg ]^{\frac{m+1}{2m p}} \\&\quad \le c \bigg [\int _0^T \bigg [ \int _\Omega |f|^{m+1} \mathrm {d}x \bigg ]^{\frac{m p}{m+1}}\mathrm {d}t \bigg ]^{\frac{m-1}{2m p}} \cdot \text{ I}^{\frac{m+1}{2m}} \\&\quad \le c\, \Vert f^m\Vert _{L^p(0,T;X)}^{\frac{m}{m+1}} \Vert f\Vert _{L^\theta (0,T;L^{\mu '}(\Omega ;\mathbb {R}^N))}^{\frac{m-1}{2(m+1)}} \Big [ \eta _1^{\frac{1}{2}} \Vert f\Vert _{L^{\theta }(0,T;L^{\mu '}(\Omega ;\mathbb {R}^N))}^{\frac{1}{2}} + M_{\eta _1}^{\frac{1}{2}} \Vert \tau _h f-f\Vert _{L^{\theta }(0,T-h;Y)}^{\frac{1}{2}} \Big ] \\&\quad \le c\, \Vert f^m\Vert _{L^p(0,T;X)}^{\frac{m}{m+1}} \Big [ 2\eta _1^{\frac{1}{2}} \Vert f\Vert _{L^{\theta }(0,T;L^{\mu '}(\Omega ;\mathbb {R}^N))}^{\frac{m}{m+1}} + \eta _1^{-\frac{(m-1)}{2(m+1)}} M_{\eta _1}^{\frac{m}{m+1}} \Vert \tau _h f-f\Vert _{L^{\theta }(0,T-h;Y)}^{\frac{m}{m+1}} \Big ]. \end{aligned}$$

We have also used the fact that $\theta =m p$ when $m>1$. Combining both cases and choosing $\eta _1$ in a suitable way, we conclude that for any $\eta _2>0$ there exists $M_{\eta _2}>0$ such that

$$\begin{aligned} \Vert \tau _h f^m&-f^m\Vert _{L^{p}(0,T-h;L^{\frac{m+1}{m}}(\Omega ;\mathbb {R}^N))} \\&\le \Vert f^m\Vert _{L^p(0,T;X)}^{\frac{m}{m+1}} \Big [ \eta _2 \Vert f\Vert _{L^{\theta }(0,T;L^{\mu '}(\Omega ;\mathbb {R}^N))}^{\frac{m}{m+1}} + M_{\eta _2} \Vert \tau _h f-f\Vert _{L^{\theta }(0,T-h;Y)}^{\frac{m}{m+1}} \Big ]. \end{aligned}$$

If $q\le \frac{m+1}{m}$, the asserted inequality follows by an application of Hölder’s inequality. In the other case where $q> \frac{m+1}{m}$, we apply Lemma 4.3 again and find that for $\eta >0$ there exists $M_{\eta }>0$ such that

$$\begin{aligned} \Vert \tau _h f^m&-f^m\Vert _{L^{p}(0,T-h;L^{q}(\Omega ;\mathbb {R}^N))} \\&\le \tfrac{1}{2} \eta \Vert \tau _h f^m-f^m\Vert _{L^{p}(0,T-h;X)} + M_{\eta } \Vert \tau _h f^m -f^m\Vert _{L^{p}(0,T-h;L^{\frac{m+1}{m}}(\Omega ;\mathbb {R}^N))} \\&\le \eta \Vert f^m\Vert _{L^{p}(0,T;X)} \\&\quad + c M_{\eta }\Vert f^m\Vert _{L^p(0,T;X)}^{\frac{m}{m+1}} \Big [ \eta _2 \Vert f\Vert _{L^{\theta }(0,T;L^{\mu '}(\Omega ;\mathbb {R}^N))}^{\frac{m}{m+1}} + M_{\eta _2} \Vert \tau _h f-f\Vert _{L^{\theta }(0,T-h;Y)}^{\frac{m}{m+1}} \Big ]. \end{aligned}$$

At this point, the claimed inequality follows by choosing $\eta _2$ so small that $cM_\eta \eta _2\le \eta$. $\square$

With these prerequisites at hand, we are able to prove the following more refined version of Theorem 4.2, where assumption (4.5) is weakened in the sense that $L^\mu (\Omega ;\mathbb {R}^N)$ is replaced by a Banach space *Y* with $X'\subset Y$.

### Theorem 4.5

Let $m\in (0,\infty )$, $p,q,\mu \in [1,\infty )$, $\theta :=\max \{1,m p\}$, $T>0$, and $\Omega \subset \mathbb {R}^n$ be a bounded domain and *X*, *Y* be Banach spaces such that $X\subset L^q(\Omega ;\mathbb {R}^N)$ and $L^{\mu '}(\Omega ;\mathbb {R}^N)\subset X'\subset Y$ with compact embeddings $X \hookrightarrow L^{q}(\Omega ;\mathbb {R}^N)$ and $L^{\mu '}(\Omega ;\mathbb {R}^N)\hookrightarrow X'$ satisfying (4.6). Moreover, let $F \subset L^{\theta }(0,T;L^{\mu '}(\Omega ;\mathbb {R}^N))$ such that $F^m\subset L^p(0,T;X)\cap L^p(0,T;L^\mu (\Omega ;\mathbb {R}^N))$. We assume that

4.9 $$\begin{aligned} F \text{ is } \text{ bounded } \text{ in } L^\theta (0,T;L^{\mu '}(\Omega ;\mathbb {R}^N)) \end{aligned}$$

and

4.10 $$\begin{aligned} F^m \text{ is } \text{ bounded } \text{ in } L^p(0,T;X). \end{aligned}$$

(i) If
  $$\begin{aligned} \Vert \tau _h f - f\Vert _{L^\theta (0,T-h;Y)}\rightarrow 0 \quad \text{ as } h\downarrow 0, \quad \text{ uniformly } \text{ for } f\in F \end{aligned}$$
  is satisfied, then $F^m$ is relatively compact in $L^{p}(0,T;L^q(\Omega ;\mathbb {R}^N))$.
(ii) If
  $$\begin{aligned} \Vert \tau _h f - f\Vert _{L^1(0,T-h;Y)}\rightarrow 0 \quad \text{ as } h\downarrow 0, \quad \text{ uniformly } \text{ for } f\in F \end{aligned}$$
  is satisfied, then $F^m$ is relatively compact in $L^{\tilde{p}}(0,T;L^q(\Omega ;\mathbb {R}^N))$ for any $\tilde{p}\in [1,p)$.

### Proof

Our aim is to apply Theorem 4.1 with $B=L^q(\Omega ;\mathbb {R}^N)$ and $F^m$ instead of *F*. From (4.10), we know that $F^m$ is bounded in $L^p(t_1,t_2;X)$ and hence in $L^1(t_1,t_2;X)$ for any $0<t_1<t_2<T$. Due to the compact embedding $X\hookrightarrow L^{q}(\Omega ;\mathbb {R}^N)$, this implies that $\{\int _{t_1}^{t_2} f^m(t) \,\mathrm {d}t: f^m\in F^m\}$ is relatively compact in $L^q(\Omega ;\mathbb {R}^N)$ for any $0<t_1<t_2<T$. Hence, (4.1) is satisfied. Next, we verify assumption (4.2). For $\eta >0$, we denote by $M_\eta >0$ the constant from Lemma 4.4. The application of the lemma yields that

$$\begin{aligned}&\Vert \tau _h f^m - f^m\Vert _{L^p(0,T-h;L^q(\Omega ;\mathbb {R}^N))} \\&\quad \le \Vert f^m\Vert _{L^p(0,T;X)}^{\frac{m}{m+1}} \bigg [ \eta \Big [\Vert f^m\Vert _{L^{p}(0,T;X)}^{\frac{1}{m+1}} + \Vert f\Vert _{L^{\theta }(0,T;L^{\mu '}(\Omega ;\mathbb {R}^N))}^{\frac{m}{m+1}} \Big ]\\&\qquad + M_{\eta } \Vert \tau _h f-f\Vert _{L^{\theta }(0,T-h;Y)}^{\frac{m}{m+1}} \bigg ] \end{aligned}$$

holds true for any $f\in F$. Assumptions (4.9) and (4.10) ensure the existence of a constant $C>0$ such that $\Vert f^m\Vert _{L^{p}(0,T;X)}\le C$ and $\Vert f\Vert _{L^{\theta }(0,T;L^{\mu '}(\Omega ;\mathbb {R}^N))}\le C$ for any $f\in F$. Hence, for $\varepsilon >0$ we may choose $\eta >0$ in the preceding inequality small enough such that

$$\begin{aligned} \Vert \tau _h f^m - f^m\Vert _{L^p(0,T-h;L^q(\Omega ;\mathbb {R}^N))} \le \varepsilon + C_{\varepsilon } \Vert \tau _h f-f\Vert _{L^{\theta }(0,T-h;Y)}^{\frac{m}{m+1}}, \end{aligned}$$

for a constant $C_\varepsilon >0$ depending on $\varepsilon$, but not on *h*. If the assumption of assertion (i) holds, then there is $h_\varepsilon$ such that if $0<h<h_\varepsilon$, then

$$\begin{aligned} \Vert \tau _h f-f\Vert _{L^{\theta }(0,T-h;Y)}^{\frac{m}{m+1}}< \varepsilon /C_\varepsilon , \end{aligned}$$

for all $f\in F$. But this means that

$$\begin{aligned} \Vert \tau _h f^m - f^m\Vert _{L^p(0,T-h;L^q(\Omega ;\mathbb {R}^N))} < 2\varepsilon \end{aligned}$$

for all $f\in F$ when $h\in (0,h_\varepsilon )$. Since $\varepsilon >0$ was arbitrary, this verifies Assumption (4.2) in Theorem 4.1. Therefore, the application of the theorem yields the compactness of $F^m$ in $L^{p}(0,T;L^q(\Omega ;\mathbb {R}^N))$ proving assertion (i).

The second assertion (ii) will follow by an interpolation argument. If $\theta =1$, then the result already follows by (i). Therefore, it is enough to consider the case $1<\theta =m p$. We consider $\tilde{p}\in [1,p)$. Without loss of generality, we may assume that $\tilde{\theta }:=m\tilde{p}>1$. We interpolate

$$\begin{aligned} \Vert \tau _h f-f\Vert _{L^{\tilde{\theta }}(0,T-h;Y)}&\le \Vert \tau _h f-f\Vert _{L^{\theta }(0,T-h;Y)}^{\frac{\theta (\tilde{\theta }-1)}{\tilde{\theta }(\theta -1)}} \Vert \tau _h f-f\Vert _{L^{1}(0,T-h;Y)}^{\frac{\theta -\tilde{\theta }}{\tilde{\theta }(\theta -1)}} \\&\le c\,\Vert f\Vert _{L^{\theta }(0,T;L^{\mu '}(\Omega ;\mathbb {R}^N))}^{\frac{\theta (\tilde{\theta }-1)}{\tilde{\theta }(\theta -1)}} \Vert \tau _h f-f\Vert _{L^{1}(0,T-h;Y)}^{\frac{\theta -\tilde{\theta }}{\tilde{\theta }(\theta -1)}}. \end{aligned}$$

Due to Assumption (4.9), $\Vert f\Vert _{L^{\theta }(0,T;L^{\mu '}(\Omega ;\mathbb {R}^N))}$ is bounded independent of *f* and therefore we have that $\Vert \tau _h f-f\Vert _{L^{\tilde{\theta }}(0,T-h;Y)}\rightarrow 0$ as $h\downarrow 0$ uniformly for $f\in F$. This allows us to apply Theorem 4.1 with $\tilde{p}$ instead of *p* and thus yields assertion (ii). $\square$

Applying Theorem 4.5 with $X=W^{1,p}_0(\Omega ;\mathbb {R}^N)$ yields the following Corollary.

### Corollary 4.6

Let $m\in (0,\infty )$, $p\in [1,\infty )$, and $T>0$, $\Omega \subset \mathbb {R}^n$ be a bounded domain and *Y* be a Banach space such that $(W^{1,p}_0(\Omega ;\mathbb {R}^N))'\subset Y$. Moreover, let $\theta :=\max \{1,m p\}$, $q\in [p,\frac{np}{n-p})$, $\mu \in [p,\frac{np}{n-p})$ if $p<n$ and $q,\mu \in [p,\infty )$ if $p\ge n$ and consider $F \subset L^{\theta }(0,T;L^{\mu '}(\Omega ;\mathbb {R}^N))$ such that $F^m\subset L^p(0,T;W^{1,p}(\Omega ;\mathbb {R}^N))$. We assume that

4.11 $$\begin{aligned} F \text{ is } \text{ bounded } \text{ in } L^\theta (0,T;L^{\mu '}(\Omega ;\mathbb {R}^N)) \end{aligned}$$

and

4.12 $$\begin{aligned} F^m \text{ is } \text{ bounded } \text{ in } L^p(0,T;W^{1,p}(\Omega ;\mathbb {R}^N)). \end{aligned}$$

(i) If
  $$\begin{aligned} \Vert \tau _h f - f\Vert _{L^\theta (0,T-h;Y)}\rightarrow 0 \quad \text{ as } h\downarrow 0, \quad \text{ uniformly } \text{ for } f\in F \end{aligned}$$
  is satisfied, then $F^m$ is relatively compact in $L^{p}(0,T;L^q(\Omega ;\mathbb {R}^N))$.
(ii) If
  $$\begin{aligned} \Vert \tau _h f - f\Vert _{L^1(0,T-h;Y)}\rightarrow 0 \quad \text{ as } h\downarrow 0, \quad \text{ uniformly } \text{ for } f\in F \end{aligned}$$
  is satisfied, then $F^m$ is relatively compact in $L^{\tilde{p}}(0,T;L^q(\Omega ;\mathbb {R}^N))$ for any $\tilde{p}\in [1,p)$.

### Proof

We restrict ourselves to the proof of case (i), since (ii) is completely analogous. Let $\widetilde{\Omega }\Subset \Omega$ and $\eta \in C_0^\infty (\Omega )$ be a nonnegative function with $\eta \equiv 1$ in $\widetilde{\Omega }$. We now consider the family of functions $F_\eta :=\{\eta ^{\frac{1}{m}}f: f\in F\}$. Then, $F_\eta \subset L^{\theta }(0,T;L^{\mu '}(\Omega ;\mathbb {R}^N))$ and $F_\eta ^m \subset L^p(0,T;W^{1,p}_0(\Omega ;\mathbb {R}^N))$. Our goal is to apply Theorem 4.5 with $X=W^{1,p}_0(\Omega ;\mathbb {R}^N)$ and $F_\eta$ in place of *F*. Note that due to the parameter ranges, all elements of *X* are $L^q$-integrable, and the inclusion $T:X \rightarrow L^q(\Omega ;\mathbb {R}^N)$ is compact. Similarly, we have a compact inclusion $\tilde{T}:X \rightarrow L^\mu (\Omega ;\mathbb {R}^N)$. Furthermore, we have an embedding $L^{\mu '}(\Omega ;\mathbb {R}^N)\hookrightarrow X'$ given by $S:=\tilde{T}'\circ J$, where $\tilde{T}': (L^\mu (\Omega ;\mathbb {R}^N))'\rightarrow X'$ is the adjoint of $\tilde{T}$, and *J* is the standard isomorphism from $L^{\mu '}(\Omega ;\mathbb {R}^N)$ to $(L^\mu (\Omega ;\mathbb {R}^N))'$. Since $\tilde{T}$ is compact, Schauder’s theorem guarantees that also *S* is compact. The condition (4.6) follows directly from the definitions of *S*, *T* and $\tilde{T}$. Notice that assumptions (4.11), (4.12) and (i) imply the corresponding assumptions in Theorem 4.5. Thus, all the assumptions of Theorem 4.5 are satisfied, and hence $F_\eta ^m$ is relatively compact in $L^{p}(0,T;L^q(\Omega ;\mathbb {R}^N))$. In particular, this implies that $F^m$ is relatively compact in $L^{p}(0,T;L^q(\widetilde{\Omega };\mathbb {R}^N))$. Since $\widetilde{\Omega }\Subset \Omega$ was arbitrary, we may conclude the relative compactness of $F^m$ in $L^{p}(0,T;L^q(\Omega ;\mathbb {R}^N))$ by a diagonal argument. $\square$

In the previous proof, we choose *X* to consist of compactly supported Sobolev functions since the corresponding space of arbitrary Sobolev functions typically is not compactly embedded into $L^q$. Such a result holds only if $\Omega$ is sufficiently regular, for example if $\Omega$ satisfies a cone condition; see [2, Theorem 6.3]. In that case, the proof is somewhat simpler.

### Remark 4.7

The assumption on the uniform convergence of the time differences in Theorem 4.6 (i) and (ii) is satisfied for functions with integrable time derivative. More precisely, [23, § 5, Lemma 4] ensures that

$$\begin{aligned} \{\partial _t f: f\in F\} \text{ is } \text{ bounded } \text{ in } L^\theta (0,T;Y) \end{aligned}$$

implies assumption (i) of Theorem 4.6 and

$$\begin{aligned} \{\partial _t f: f\in F\} \text{ is } \text{ bounded } \text{ in } L^1(0,T;Y) \end{aligned}$$

implies assumption (ii) of Theorem 4.6.

## The approximation scheme

For $k>1$, we approximate the vector field *A* defined in (2.5) by vector fields $A_k$ given by

5.1 $$\begin{aligned} A_k(v,\xi ):= A\big (T_k(v),\beta T_k(v)^{\beta -1}\xi \big ) = {\left\{ \begin{array}{ll} A(\frac{1}{k},\beta \frac{1}{k^{\beta -1}}\xi ), \quad v<\frac{1}{k}\\ A(v,\beta v^{\beta -1}\xi ), \quad \frac{1}{k}\le v \le k\\ A(k, \beta k^{\beta -1}\xi ), \quad v > k, \end{array}\right. } \end{aligned}$$

for $v\in \mathbb {R}$ and $\xi \in \mathbb {R}^n$, where the truncation $T_k:\mathbb {R}\rightarrow \mathbb {R}$ is defined by

5.2 $$\begin{aligned} T_k(s):= \min \big \{ k, \max \{ s, \tfrac{1}{k}\}\big \}. \end{aligned}$$

Exploiting the definition of *A*, we can express $A_k$ as

5.3 $$\begin{aligned} A_k(v,\xi )&= T_k(v)^\alpha |\xi +\nabla z|^{p-2}(\xi +\nabla z) \nonumber \\&= {\left\{ \begin{array}{ll} k^{-\alpha }|\xi +\nabla z|^{p-2}(\xi +\nabla z), \quad &{} v<\frac{1}{k}\\ v^\alpha |\xi +\nabla z |^{p-2}(\xi + \nabla z), &{} \frac{1}{k}\le v \le k\\ k^\alpha |\xi +\nabla z|^{p-2}(\xi +\nabla z), &{} v > k. \end{array}\right. } \end{aligned}$$

### Properties of $A_k$

Using the properties of the vector field corresponding to the *p*-Laplace operator, we can verify the following useful basic properties of $A_k$.

#### Monotonicity

Due to Remark 3.4, we know that there exists a constant *c* depending only on *p* such that

5.4 $$\begin{aligned}&(A_k(v,\xi )-A_k(v,\eta ))\cdot (\xi -\eta )\nonumber \\&\quad \ge {\left\{ \begin{array}{ll} c\, k^{-\alpha }|\xi -\eta |^p, &{}p\ge 2 \\ c\, k^{-\alpha }\big (|\xi +\nabla z|^2+|\eta +\nabla z|^2\big )^{\frac{p-2}{2}}|\xi -\eta |^2, &{} p<2, \end{array}\right. } \end{aligned}$$

holds true for any $v\in \mathbb {R}$ and $\xi ,\eta \in \mathbb {R}^n$. If $p<2$ and $\xi \equiv \eta \equiv -\nabla z$, then the right-hand side has to be interpreted as zero.

#### Boundedness

For any $v\in \mathbb {R}$ and $\xi \in \mathbb {R}^n$, we have

5.5 $$\begin{aligned} |A_k(v,\xi )|\le k^\alpha |\xi +\nabla z|^{p-1}\le 2^{p-1}k^\alpha \big (|\xi |^{p-1}+|\nabla z|^{p-1}\big ). \end{aligned}$$

#### Coercivity

Due to the definition of $A_k$ and Lemma 3.5, we have that

5.6 $$\begin{aligned} A_k(v,\xi )\cdot \xi&= T_k(v)^\alpha |\xi +\nabla z|^{p-2}(\xi +\nabla z)\cdot \xi \nonumber \\&\ge T_k(v)^\alpha \big [2^{-p}|\xi |^p - 2^p|\nabla z|^p\big ] \nonumber \\&\ge 2^{-p}k^{-\alpha } |\xi |^p - 2^pk^\alpha |\nabla z|^p \end{aligned}$$

for any $v\in \mathbb {R}$ and $\xi \in \mathbb {R}^n$.

### Weak solutions of the approximating equation

In this section, we want to find weak solutions to the approximating problems

5.7 $$\begin{aligned} \left\{ \begin{array}{ll} \partial _t v_k - \nabla \cdot A_k(v_k, \nabla v_k) = f-k^{-\alpha }\nabla \cdot \big (|\nabla z|^{p-2}\nabla z\big ) &{}\quad \text { in } \Omega _T,\\ v_k = \frac{1}{k} &{} \quad \text { on } \partial \Omega \times (0,T),\\ v_k(\cdot ,0) = \frac{1}{k}+\Psi &{} \quad \text { in } \overline{\Omega }, \end{array} \right. \end{aligned}$$

where $\Psi =\psi -z$. By formally integrating by parts, we are led to the following definition.

#### Definition 5.1

A function $v_k\in C([0,T]; L^2(\Omega ))\cap \frac{1}{k}+ L^p(0,T; W^{1,p}_0(\Omega ))$ is an admissible weak solution to the Cauchy–Dirichlet problem (5.7) if

5.8 $$\begin{aligned} \iint _{\Omega _T} \big [A_k(v_k,\nabla v_k)\cdot \nabla \varphi - v_k\partial _t\varphi \big ]\mathrm {d}x\mathrm {d}t = \iint _{\Omega _T} \big [f\varphi + k^{-\alpha }|\nabla z|^{p-2}\nabla z\cdot \nabla \varphi \big ] \mathrm {d}x \mathrm {d}t \end{aligned}$$

for all $\varphi \in C^\infty _0(\Omega _T)$ and $v_k(\cdot ,0)=\tfrac{1}{k}+\Psi$ in $\Omega$.

Using a test function of the form $[\varphi (x,t)\xi (t)]_{\bar{h}}$ where $\varphi \in C^\infty (\bar{\Omega }\times [0,T])$ vanishes if *x* is outside a compact subset of $\Omega$ and $\xi$ is any smooth function compactly supported in $(0,T-h)$, one easily verifies that any solution $v_k$ in the sense of Definition 5.1 satisfies the following equation with Steklov means $[\,\cdot \,]_h$ defined in (3.2):

5.9 $$\begin{aligned}&\int ^b_a\int _\Omega \Big [\partial _t [v_k]_{h} \varphi + \big [A_k(v_k,\nabla v_k)\big ]_{h} \cdot \nabla \varphi \Big ] \mathrm {d}x \mathrm {d}t\nonumber \\&\qquad = \int ^b_a\int _\Omega \big [[f]_{h} \varphi + k^{-\alpha }|\nabla z|^{p-2}\nabla z\cdot \nabla \varphi \big ]\mathrm {d}x \mathrm {d}t, \end{aligned}$$

for all $0\le a < b \le T-h$. In fact, by approximation with smooth functions, one sees that all $\varphi \in L^p(0,T;W^{1,p}_0(\Omega ))\cap L^\infty (\Omega _T)$ are admissible in (5.9). An analogous identity holds true for the Steklov averages $[\,\cdot \,]_{\bar{h}}$ and $h \le a < b \le T$.

We now prove the existence of a solution to the regularized problem in the sense of Definition 5.1. We will follow the functional analytic approach of Showalter [22] making use of Galerkin’s method. In fact, one can reason as in the proof of [22, Theorem 4.1, Sect. III.4], despite the somewhat weaker coercivity condition in our case. For the reader’s convenience, we present the full argument below. We have opted to avoid the theory of operators of type M used by Showalter, exploiting instead the stronger monotonicity property of the vector field $A_k$.

#### Lemma 5.2

For any $k>1$, there exists at least one admissible weak solution $v_k$ to (5.7) in the sense of Definition 5.1.

#### Proof

We fix $k>1$ and consider the modified vector field

$$\begin{aligned} \tilde{A}_k(w,\xi ):=A_k\big (\tfrac{1}{k} + w, \xi \big )= T_k(\tfrac{1}{k} + w )^\alpha |\xi + \nabla z|^{p-2}(\xi + \nabla z), \end{aligned}$$

for $w\in \mathbb {R}$ and $\xi \in \mathbb {R}^n$. We prove the existence of a function $w\in C([0,T];L^2(\Omega ))\cap L^p(0,T;W^{1,p}_0(\Omega ))$ satisfying

5.10 $$\begin{aligned} \iint _{\Omega _T}\big [\tilde{A}_k(w,\nabla w)\cdot \nabla \varphi - w\partial _t\varphi \big ]\mathrm {d}x\mathrm {d}t = \iint _{\Omega _T} \big [f\varphi + k^{-\alpha }|\nabla z|^{p-2}\nabla z\cdot \nabla \varphi \big ] \mathrm {d}x \mathrm {d}t, \end{aligned}$$

for all $\varphi \in C^\infty _0(\Omega _T)$, and $w(\cdot ,0)= \Psi$ in $\Omega$. Then, $v_k:= \tfrac{1}{k} + w$ is an admissible weak solution in the sense of Definition 5.1.

We denote $V=L^2(\Omega )\cap W^{1,p}_0(\Omega )$ and define $\mathcal {A}: V\rightarrow V'$ by

$$\begin{aligned} \langle \mathcal {A}(w), v\rangle := \int _\Omega \tilde{A}_k(w,\nabla w)\cdot \nabla v \mathrm {d}x, v,w\in V. \end{aligned}$$

We define $F\in L^{p'}(0,T;V')$ by setting

$$\begin{aligned} \langle F(t), v\rangle := \int _\Omega \big [f(\cdot ,t) v + k^{-\alpha }|\nabla z|^{p-2}\nabla z\cdot \nabla v\big ] \mathrm {d}x, \;\; v\in V. \end{aligned}$$

Recall that we have the inclusions $V \hookrightarrow L^2(\Omega )\hookrightarrow V'$ with *V* being dense in $L^2(\Omega )$. Then, (5.10) is equivalent to

$$\begin{aligned} w'+\mathcal {A}(w)=F, \quad \text {in } V'. \end{aligned}$$

This equation can be understood in the weak sense using the Bochner integral, or equivalently pointwise a.e. Pick a basis $(v_j)^{\infty }_{j=1}$ of *V*, and for each $m\in \mathbb {N}$ vectors $\psi _m \in \text {span} (v_1,\dots ,v_m) =:V_m$ converging to $\Psi$ in $L^2(\Omega )$, and consider for a fixed $m\in \mathbb {N}$ the problem of finding a map $w_m:[0,T]\rightarrow V_m$ satisfying

5.11 $$\begin{aligned} \left\{ \begin{array}{l} (w_m'(t),v_j)+\langle \mathcal {A}(w_m(t)),v_j\rangle = \langle F(t),v_j\rangle , \quad \text{ for } j\in \{1,\dots m\} \text{ and } \text{ a.e. } t.\\ w_m(0)=\psi _m \end{array} \right. \end{aligned}$$

Here, $(\cdot ,\cdot )$ denotes the inner product in $L^2(\Omega )$ and $\langle \cdot ,\cdot \rangle$ denotes the dual pairing of $V'$ and *V*. We define $g:\mathbb {R}^m\rightarrow \mathbb {R}^m$ with components

$$\begin{aligned} g^i(y)=-\langle \mathcal {A}(\Sigma ^m_{j=1}y^j v_j), v_i\rangle , \quad \text{ for } y\in \mathbb {R}^m \end{aligned}$$

and $\tilde{f}:[0,T]\rightarrow \mathbb {R}^m$ with components

$$\begin{aligned} \tilde{f}^i(t)=\langle F(t),v_i\rangle . \end{aligned}$$

The dominated convergence theorem shows that *g* is continuous, $\tilde{f}$ is evidently integrable and the matrix with components $(v_i,v_j)$, $i,j\in \{1,\dots ,m\}$ is invertible. Therefore, Lemma 3.9 guarantees that the problem (5.11) has a solution $w_m$ on some interval $[0,\delta ]$. This solution can be extended to a maximal interval $J\subset [0,T]$. Multiplying (5.11) by the component function $w_m^j(t)$ of $w_m$ in the basis $(v_j)^m_{j=1}$ and summing over *j*, we have

5.12 $$\begin{aligned} (w_m'(t),w_m(t))+\langle \mathcal {A}(w_m(t)),w_m(t)\rangle = \langle F(t),w_m(t)\rangle \text { for a.e. } t\in J. \end{aligned}$$

The coercivity property (5.6) of $A_k$ implies the same property for $\tilde{A}_k$, from which we obtain for all $v\in V$ that

$$\begin{aligned} \langle \mathcal {A}(v),v\rangle&\ge 2^{-p}k^{-\alpha }\int _\Omega |\nabla v|^p\mathrm {d}x - 2^p k^{\alpha }\int _\Omega |\nabla z|^p\mathrm {d}x \\&\ge \tfrac{1}{c} \Vert v\Vert ^p_{W^{1,p}(\Omega )} - c\Vert \nabla z\Vert ^p_{L^p(\Omega )}, \end{aligned}$$

with a constant $c> 1$ depending on $\alpha , p, k$ and $\Omega$. Using this estimate in (5.12) shows that

$$\begin{aligned}&(w_m'(t),w_m(t)) + \tfrac{1}{c}\Vert w_m(t)\Vert ^p_{W^{1,p}(\Omega )}\\&\quad \le c\Vert \nabla z\Vert ^p_{L^p(\Omega )} + \Vert F(t)\Vert _{W^{-1,p'}(\Omega )}\Vert w_m(t)\Vert _{W^{1,p}(\Omega )}, \end{aligned}$$

for a.e. $t\in J$. Here, we have extended *F*(*t*) to an element of $W^{-1,p'}(\Omega )=(W^{1,p}_0(\Omega ))'$ using the same formula as before. Applying Young’s inequality to the last term, we find that

$$\begin{aligned} (w_m'(t),w_m(t)) + \tfrac{1}{c}\Vert w_m(t)\Vert ^p_{W^{1,p}(\Omega )} \le c\Vert \nabla z\Vert ^p_{L^p(\Omega )} + c\Vert F(t)\Vert _{W^{-1,p'}(\Omega )}^{p'}, \end{aligned}$$

where $c>1$ depends on $\alpha , p, k$ and $\Omega$. Integrating the last inequality, we have

5.13 $$\begin{aligned} \tfrac{1}{2}\Vert w_m(t)\Vert _{L^2(\Omega )}^2&+ \tfrac{1}{c}\int ^t_0 \Vert w_m(s)\Vert ^p_{W^{1,p}(\Omega )} \mathrm {d}s \nonumber \\&\le c\Vert \nabla z\Vert ^p_{L^p(\Omega )} T + \tfrac{1}{2}\Vert \psi _m\Vert _{L^2(\Omega )}^2 + c\int ^T_0 \Vert F(s)\Vert _{W^{-1,p'}(\Omega )}^{p'} \mathrm {d}s, \end{aligned}$$

for all $$t\in J$$. This shows that $$\Vert w_m\Vert _{L^2(\Omega )}$$ and the component functions $$w^i_m$$ stay bounded on *J*, and from the system of equations we conclude that $$w_m$$ is absolutely continuous on all of *J*. If *J* is not the interval [0, *T*], then $$J=[0,b)$$ where $$b<T$$. Then, the uniform continuity of $$w_m$$ and the finite dimension of $$V_m$$ show that there is a limit of $$w_m(t)$$ as $$t\uparrow b$$ which allows us to extend $$w_m$$, thus contradicting maximality. Hence, $$w_m$$ must indeed be defined on all of [0, *T*]. Moreover, since $$\psi _m\rightarrow \Psi $$ in $$L^2(\Omega )$$, the estimate (5.13) shows on the one hand that $$(w_m)$$ is a bounded sequence in $$L^\infty (0,T;L^2(\Omega ))$$ and on the other hand that $$(w_m)$$ is a bounded sequence in $$L^p(0,T;W^{1,p}(\Omega ))$$. Hence, we infer that $$w_m$$ is a bounded sequence in $$L^p(0,T;V)$$. From the definition of $$\mathcal {A}$$ and (5.5), we see that

$$\begin{aligned} \Vert \mathcal {A}(v)\Vert _{V'}\le c\Vert v\Vert ^{p-1}_{V}+c\Vert \nabla z\Vert ^{p-1}_{L^p(\Omega )}, \end{aligned}$$

with $c=c(\alpha ,p,k)$, and hence $(\mathcal {A}(w_m))$ is a bounded sequence in $L^{p'}(0,T;V')$. By reflexivity, we have a subsequence still labeled as $(w_m)$ which converges weakly to $w\in L^p(0,T;V)$ and for which $(\mathcal {A}(w_m))$ converges weakly to $\xi \in L^{p'}(0,T;V')$. Furthermore, (5.13) shows that $(w_m(T))$ is bounded in $L^2(\Omega )$, so we may assume that $(w_m(T))$ converges weakly to some $w^*\in L^2(\Omega )$. Take now $\varphi \in C^\infty ([0,T])$ and $v\in V_m$. From (5.11), we see that

$$\begin{aligned} (w_m'(t),\varphi (t)v)+\langle \mathcal {A}(w_m(t)),\varphi (t)v\rangle&= \langle F(t),\varphi (t)v\rangle . \end{aligned}$$

Integrating this identity, we obtain

$$\begin{aligned} -\int ^T_0&(w_m(t),v)\varphi '(t)\mathrm {d}t + \int ^T_0 \langle \mathcal {A}(w_m(t)),\varphi (t)v\rangle \mathrm {d}t\\&= (\psi _m,v)\varphi (0) - (w_m(T),v)\varphi (T) +\int ^T_0 \langle F(t),\varphi (t)v\rangle \mathrm {d}t. \end{aligned}$$

Due to the weak convergences mentioned above, we obtain by taking $m\rightarrow \infty$ that

5.14 $$\begin{aligned} -\int ^T_0&(w(t),v)\varphi '(t)\mathrm {d}t + \int ^T_0 \langle \xi (t),v\rangle \varphi (t) \mathrm {d}t\nonumber \\&= (\Psi ,v)\varphi (0) - (w^*,v)\varphi (T)+ \int ^T_0 \langle F(t),\varphi (t)v\rangle \mathrm {d}t, \end{aligned}$$

for all $v\in V_{m_o}$ for any $m_o\in \mathbb {N}$, and by approximation for all $v\in V$. This shows (by taking $\varphi \in C^\infty _0(0,T)$) that

5.15 $$\begin{aligned} w' + \xi = F, \end{aligned}$$

in $L^{p'}(0,T;V')$, and thus $w\in C([0,T];L^2(\Omega ))$; see also Proposition 1.2 of Sect. III.1 in [22]. Next, we show that *w* satisfies the right initial condition. Using the test function

$$\begin{aligned} \varphi (t)={\left\{ \begin{array}{ll} \tfrac{1}{\varepsilon }(\varepsilon -t), &{}t\in [0,\varepsilon ], \\ 0, &{}t>\varepsilon , \end{array}\right. } \end{aligned}$$

in (5.14) we have for all $v\in V$,

$$\begin{aligned} \bigg (\frac{1}{\varepsilon }\int ^\varepsilon _0 w(t)\mathrm {d}t-\Psi , v \bigg )=\int ^\varepsilon _0 \langle F(t) - \xi (t),v\rangle \varphi (t)\mathrm {d}t, \end{aligned}$$

where the integral on the left-hand side is taken in the Bochner sense of *w* as an $L^2(\Omega )$-valued map. By the density of *V* in $L^2(\Omega )$, this implies

$$\begin{aligned} \bigg \Vert \frac{1}{\varepsilon }\int ^\varepsilon _0 w(t)\mathrm {d}t-\Psi \bigg \Vert _{L^2(\Omega )}\le \int ^\varepsilon _0 \Vert F(t) - \xi (t)\Vert _{V'} \mathrm {d}t. \end{aligned}$$

The right-hand side converges to zero as $\varepsilon \downarrow 0$. On the other hand, since $w\in C([0,T];L^2(\Omega ))$, we know that the limit of the integral average appearing on the left-hand side is *w*(0). Thus, we have confirmed that $w(0)=\Psi$. It only remains to show that $\xi = \mathcal {A}(w)$. Since $\xi$ is the weak limit of $(\mathcal {A}(w_m))$, it is sufficient to show that $(\mathcal {A}(w_m))$ converges weakly to $\mathcal {A}(w)$. In order to prove the weak convergence, we first show the $L^p$-convergence of $(\nabla w_m)$ to $\nabla w$. From the monotonicity condition (5.4) satisfied by $A_k$, it follows that

$$\begin{aligned} \langle \mathcal {A}w_m - \mathcal {A}w, w_m-w\rangle \ge {\left\{ \begin{array}{ll} c \displaystyle {\iint _{\Omega _T} |\nabla w_m - \nabla w|^p \mathrm {d}x \mathrm {d}t}, &{}p\ge 2\\ c \displaystyle {\iint _{\Omega _T\cap \{\nabla w \ne \nabla w_m\}}W_m^{p-2}|\nabla w_m-\nabla w|^2\mathrm {d}x \mathrm {d}t}, &{}p<2, \end{array}\right. } \end{aligned}$$

where $$c=c(p,k)$$ and

$$\begin{aligned} W_m:=|\nabla w_m + \nabla z| + |\nabla w+\nabla z|. \end{aligned}$$

In the case $p<2$, by Hölder’s inequality we may estimate

$$\begin{aligned} \iint _{\Omega _T}&|\nabla w_m - \nabla w|^p \mathrm {d}x \mathrm {d}t \\&= \iint _{\Omega _T\cap \{\nabla w \ne \nabla w_m\}}|\nabla w_m - \nabla w|^p W_m^{\frac{p(p-2)}{2}}W_m^{\frac{p(2-p)}{2}}\mathrm {d}x \mathrm {d}t\\&\le \bigg [\iint _{\Omega _T\cap \{\nabla w \ne \nabla w_m\}}W_m^{p-2}|\nabla w_m-\nabla w|^2\mathrm {d}x\mathrm {d}t\bigg ]^\frac{p}{2}\bigg [\iint _{\Omega _T}W_m^p\mathrm {d}x\mathrm {d}t\bigg ]^\frac{2-p}{2}. \end{aligned}$$

The last factor is bounded independently of *m* since $(w_m)$ is bounded in $L^p(0,T;V)$. Thus, setting $\nu =\max \{1,\tfrac{2}{p}\}$ we have in any case that

$$\begin{aligned}&\bigg [\iint _{\Omega _T} |\nabla w_m - \nabla w|^p \mathrm {d}x \mathrm {d}t\bigg ]^\nu \\&\quad \le c\int _0^T\big \langle \mathcal {A}(w_m) - \mathcal {A}(w), w_m-w\big \rangle \mathrm {d}t \\&\quad = c\int _0^T\big [\langle \mathcal {A}(w_m), w_m\rangle - \langle \mathcal {A}(w_m), w\rangle -\langle \mathcal {A}(w), w_m - w\rangle \big ]\mathrm {d}t \\&\quad = c\int _0^T \big [\langle F, w_m\rangle - \langle \mathcal {A}(w_m), w\rangle -\langle \mathcal {A}(w), w_m - w\rangle \big ] \mathrm {d}t \\&\qquad + \tfrac{1}{2}\Vert \psi _m\Vert ^2_{L^2(\Omega )} - \tfrac{1}{2}\Vert w_m(T)\Vert ^2_{L^2(\Omega )}, \end{aligned}$$

for a constant *c* independent of *m*. In the last step, we have used (5.12) integrated over [0, *T*]. The weak convergences of $(w_m)$ to *w* and $(\mathcal {A}(w_m))$ to $\xi$, the norm convergence of $(\psi _m)$, and the weak lower semicontinuity of the norm applied to the term $\Vert w_m(T)\Vert ^2_{L^2(\Omega )}$ then show that

$$\begin{aligned} \limsup _{m\rightarrow \infty }&\bigg [\iint _{\Omega _T} |\nabla w_m - \nabla w|^p \mathrm {d}x \mathrm {d}t\bigg ]^\nu \\&\le c\int _0^T\big [\langle F, w\rangle - \langle \xi , w\rangle \big ]\mathrm {d}t + \tfrac{1}{2}\Vert \Psi \Vert ^2_{L^2(\Omega )} - \tfrac{1}{2}\Vert w^*\Vert ^2_{L^2(\Omega )} = 0, \end{aligned}$$

where in the last step we have used (5.15) applied to *w* and integrated over [0, *T*]. Thus, we have obtained the desired $L^p$-convergence of $\nabla w_m$ to $\nabla w$. To see that the weak convergence of $\mathcal {A}(w_m)$ to $\mathcal {A}(w)$ follows from this, we use Lemma 3.6 and obtain

$$\begin{aligned}&|\langle \mathcal {A}(w_m) -\mathcal {A}(w), v\rangle | \\&\qquad \le c\iint _{\Omega _T} \big | |\nabla w_m + \nabla z|^{p-2}(\nabla w_m + \nabla z) - |\nabla w + \nabla z|^{p-2}(\nabla w + \nabla z)\big | |\nabla v|\mathrm {d}x \mathrm {d}t\\&\qquad \le c\iint _{\Omega _T}\big (|\nabla w + \nabla z| + |\nabla w_m-\nabla w|\big )^{p-2}|\nabla w_m - \nabla w| |\nabla v| \mathrm {d}x \mathrm {d}t\\&\qquad \le c \iint _{\Omega _T} \big (|\nabla w_m -\nabla w|^{p-1} + b_p|\nabla w + \nabla z|^{p-2}|\nabla w_m -\nabla w|\big )|\nabla v| \mathrm {d}x \mathrm {d}t, \end{aligned}$$

where $b_p = 0$ if $p<2$ and $b_p=1$ if $p\ge 2$. Hölder’s inequality and the $L^p$-convergence of $\nabla w_m$ show that the last expression converges to zero as $m \rightarrow \infty$, so we have confirmed that $\mathcal {A}(w) = \xi$. $\square$

### Properties of the approximating solutions

In this section, we investigate the properties of the approximating solutions obtained in Lemma 5.2. We prove lower bounds for the solutions $v_k$ in terms of *k* and upper bounds which are independent of *k*. Moreover, we obtain a uniform bound for the $L^p(\Omega _T)$-norms of the gradients $\nabla v_k^\beta$.

#### Lemma 5.3

Let $k>1$ and $v_k$ be an admissible weak solution to the Cauchy–Dirichlet problem (5.7) in the sense of Definition 5.1. Then, we have $v_k\ge \tfrac{1}{k}$ a.e. in $\Omega _T$.

#### Proof

We use a comparison principle argument. Consider the version of (5.9) with Steklov means $[\,\cdot \,]_{\bar{h}}$, that is

$$\begin{aligned}&\int ^b_a\int _\Omega \Big [\partial _t [v_k]_{\bar{h}}\varphi + [A_k(v_k,\nabla v_k)]_{\bar{h}}\cdot \nabla \varphi \Big ] \mathrm {d}x \mathrm {d}t \\&\quad = \int ^b_a \int _\Omega \big [[f]_{\bar{h}}\varphi + k^{-\alpha }|\nabla z|^{p-2}\nabla z\cdot \nabla \varphi \big ]\mathrm {d}x \mathrm {d}t, \end{aligned}$$

for $h<a<b<T$ and the test function $\varphi =H_\delta \left( \tfrac{1}{k}-v_k\right)$, where

$$\begin{aligned} H_\delta (s):={\left\{ \begin{array}{ll} 0, \quad &{}s<0 \\ \frac{s}{\delta }, &{}s\in [0,\delta ] \\ 1, &{} s>\delta . \end{array}\right. } \end{aligned}$$

The test function is admissible since $v_k-\tfrac{1}{k}\in L^p(0,T; W^{1,p}_0(\Omega ))$, and $H_\delta$ and $H_\delta '$ are bounded. We define $G_\delta$ as

$$\begin{aligned} G_\delta (s):=\int ^s_0 H_\delta (\sigma )\mathrm {d}\sigma = {\left\{ \begin{array}{ll} 0, \quad &{}s<0 \\ \frac{s^2}{2\delta }, &{}s\in [0,\delta ] \\ s-\frac{\delta }{2}, &{}s>\delta . \end{array}\right. } \end{aligned}$$

By the properties of the Steklov average and the convexity of $G_\delta$, we have

$$\begin{aligned} \partial _t\big [G_\delta \left( \tfrac{1}{k}-v_k\right) \big ]_{\bar{h}}(x,t)&=\tfrac{1}{h}\big [G_\delta \left( \tfrac{1}{k}-v_k\right) (x,t)-G_\delta \left( \tfrac{1}{k}-v_k\right) (x,t-h)\big ] \\&\le \tfrac{1}{h} H_\delta \left( \tfrac{1}{k}-v_k\right) (x,t)\big [ \left( \tfrac{1}{k}-v_k\right) (x,t) - \left( \tfrac{1}{k}-v_k\right) (x,t-h)\big ] \\&= -\tfrac{1}{h}H_\delta \left( \tfrac{1}{k}-v_k\right) (x,t)\big [v_k(x,t)-v_k(x,t-h)\big ] \\&= -\varphi (x,t) \partial _t[v_k]_{\bar{h}}(x,t). \end{aligned}$$

Using this estimate and the fact that $f\ge 0$ in the above identity, we obtain

$$\begin{aligned} \int ^b_a\int _\Omega \Big [\partial _t\big [G_\delta \left( \tfrac{1}{k}-v_k\right) \big ]_{\bar{h}} + \big [k^{-\alpha }|\nabla z|^{p-2}\nabla z - [A_k(v_k,\nabla v_k)]_{\bar{h}}\big ]\cdot \nabla \varphi \Big ]\mathrm {d}x \mathrm {d}t \le 0. \end{aligned}$$

Taking $h\downarrow 0$, we find that

5.16 $$\begin{aligned} \bigg [\int _\Omega G_\delta \left( \tfrac{1}{k}-v_k\right) \mathrm {d}x\bigg ]^b_a + \int ^b_a\int _\Omega \big [k^{-\alpha }|\nabla z|^{p-2}\nabla z - A_k(v_k,\nabla v_k)\big ] \cdot \nabla \varphi \mathrm {d}x \mathrm {d}t \le 0 \end{aligned}$$

holds true for a.e. $0<a<b<T$. Note that

$$\begin{aligned} \nabla \varphi = -H'_\delta \left( \tfrac{1}{k}-v_k\right) \nabla v_k=-\delta ^{-1}\chi _{\{0<\frac{1}{k}-v_k<\delta \}}\nabla v_k= -\delta ^{-1}\chi _{\{\frac{1}{k}-\delta< v_k < \frac{1}{k}\}}\nabla v_k. \end{aligned}$$

Thus, whenever the second integrand is nonzero we have $v_k<\tfrac{1}{k}$ and then

$$\begin{aligned}&-\Big [k^{-\alpha }|\nabla z|^{p-2}\nabla z - A_k(v_k,\nabla v_k)\Big ] \cdot \nabla v_k \\&\qquad = k^{-\alpha }\Big [ |\nabla v_k+\nabla z|^{p-2}(\nabla v_k+\nabla z)-|\nabla z|^{p-2}\nabla z\Big ]\cdot (\nabla v_k+\nabla z -\nabla z)\ge 0. \end{aligned}$$

This shows that the second integral of (5.16) is nonnegative so we can drop it. Thus, we end up with

$$\begin{aligned} \int _\Omega G_\delta \left( \tfrac{1}{k}-v_k\right) (x,b)\mathrm {d}x\le \int _\Omega G_\delta \left( \tfrac{1}{k}-v_k\right) (x,a)\mathrm {d}x \end{aligned}$$

for a.e. $0<a<b<T$. Since $v_k\in C([0,T];L^2(\Omega ))$ and $v_k(0)=\frac{1}{k}+ \Psi$, we obtain, by taking $a\downarrow 0$ that

$$\begin{aligned} \int _\Omega G_\delta \left( \tfrac{1}{k}-v_k\right) (x,b)\mathrm {d}x\le \int _\Omega G_\delta (-\Psi ) (x)\mathrm {d}x = 0, \end{aligned}$$

where the last equality follows from the fact that $\Psi \ge 0$. Taking the limit $\delta \rightarrow 0$, we obtain

$$\begin{aligned} \int _\Omega \left( \tfrac{1}{k}-v_k\right) _+(x,b)\mathrm {d}x \le 0, \end{aligned}$$

for a.e. $b \in (0,T)$. Thus, $v_k\ge \frac{1}{k}$ a.e. in $\Omega _T$. $\square$

We now intend to show that the approximative solutions $v_k$ are bounded in the $L^\infty$-norm by a constant independent of *k*. In the proof, we will make use of the truncation defined in (5.2) and the function

5.17 $$\begin{aligned} \tilde{v}_k:=T_k\circ v_k. \end{aligned}$$

Note that for $s\ge \frac{1}{k}$, we have $T_k(s)=\min \{s,k\}$ and hence $\tilde{v}_k=\min \{v_k,k\}$ by Lemma 5.3. The proof is divided into three steps. First, we establish an energy estimate for $\tilde{v}_k$. We use this result to show that the $L^{\beta p}$-norm of $\tilde{v}_k$ is bounded independently of *k*. Finally, the energy estimate is utilized in a De Giorgi-type iteration to obtain a bound in terms of the $L^{\beta p}$-norm of $\tilde{v}_k$, which by the previous observation concludes the proof.

#### Lemma 5.4

Let $k>1$ and $v_k$ be an admissible weak solution to the Cauchy–Dirichlet problem (5.7) in the sense of Definition 5.1, and let $M\ge \sup _{\bar{\Omega }}\Psi +1$. Then, the function $\tilde{v}_k$ defined in (5.17) satisfies

5.18 $$\begin{aligned} \sup _{\tau \in [0,T]} \int _\Omega&\big ( \tilde{v}_k^{\frac{\beta +1}{2}}-M^{\frac{\beta +1}{2}}\big )^2_+(x,\tau )\mathrm {d}x + \iint _{\Omega _T} \big |\nabla (\tilde{v}_k^\beta -M^\beta )_+\big |^p \mathrm {d}x\mathrm {d}t \nonumber \\&\le c \iint _{\Omega _T \cap \{\tilde{v}_k>M\}} \big [|\nabla z|^{\beta p}+ f^{p'} + M^{\beta p}\big ]\mathrm {d}x\mathrm {d}t, \end{aligned}$$

for a constant $c=c(\beta ,p,\Omega )$ which does not depend on *k*.

#### Proof

In the case $k\le M$, the inequality trivially holds, since $\tilde{v}_k\le k\le M$. Therefore, we are left with the case $k> M$. For $0<\varepsilon<\tau<\tau +\varepsilon <T$ and $\zeta _{\tau ,\varepsilon }$ we define

$$\begin{aligned} \zeta _{\tau ,\varepsilon }(t)={\left\{ \begin{array}{ll} 0, \quad &{} t<0 \\ \frac{1}{\varepsilon }t, &{} t\in [0,\varepsilon ] \\ 1, \quad &{}t\in [\varepsilon , \tau ] \\ 1-\frac{1}{\varepsilon }(t-\tau ), &{}t\in [\tau ,\tau +\varepsilon ] \\ 0, &{} t>\tau +\varepsilon . \end{array}\right. } \end{aligned}$$

We use the mollified formulation (5.9) of the differential equation with $a=0$, $b=T-h$ and $\varphi =\zeta _{\tau ,\varepsilon }\big (T_k([v_k]_h)^\beta -M^\beta \big )_+$. We take *h* so small that the factor $\zeta _{\tau ,\varepsilon }$ is supported in $[0,T-h]$. Then, the boundedness of $T_k([v_k]_h)$ and the chain rule imply that $\varphi \in L^p(0,T;W^{1,p}_0(\Omega ))\cap L^\infty (\Omega _T)$, which means that $\varphi$ is admissible as a test function.

Our goal is to pass to the limit $h\downarrow 0$ in the mollified differential equation, possibly replacing the equality by a suitable estimate. For the elliptic term, we conclude that

$$\begin{aligned}&\lim _{h\downarrow 0}\iint _{\Omega _{T-h}}[A_k(v_k,\nabla v_k)]_h\cdot \nabla \varphi \mathrm {d}x \mathrm {d}t \\&\quad = \iint _{\Omega _T} \zeta _{\tau ,\varepsilon } A_k(v_k,\nabla v_k)\cdot \nabla \big (\tilde{v}_k^\beta -M^\beta \big )_+ \mathrm {d}x\mathrm {d}t. \end{aligned}$$

The convergence of $\nabla \varphi$ in the $L^p$-norm can be seen from the chain rule, and the fact that the outer function $s\mapsto (T_k(s)^\beta -M^\beta )_+$ is piecewise $C^1$ with bounded derivative. We now introduce the abbreviation

$$\begin{aligned} g(s):=&\,\big (T_k(s)^\beta -M^\beta \big )_+, G(s):=\int ^s_0 g(t)\mathrm {d}t\\ =&\,{\left\{ \begin{array}{ll} 0, \quad &{}s\le M \\ \mathfrak {b}[s,M], &{}s\in [M,k] \\ \mathfrak {b}[k,M]+(k^\beta -M^\beta )(s-k), &{}s>k \end{array}\right. } \end{aligned}$$

and observe that $\varphi =\zeta _{\tau ,\varepsilon }g([v_k]_h)$. Recall that the boundary term $\mathfrak {b}$ has been introduced in (3.1). This allows us to treat the parabolic term as

$$\begin{aligned}&\iint _{\Omega _{T-h}} \partial _t [v_k]_h \varphi \mathrm {d}x \mathrm {d}t \\&\quad = \iint _{\Omega _{T-h}} \zeta _{\tau ,\varepsilon } \partial _t [v_k]_h g([v_k]_h) \mathrm {d}x \mathrm {d}t = \iint _{\Omega _{T-h}} \zeta _{\tau ,\varepsilon } \partial _t G([v_k]_h) \mathrm {d}x \mathrm {d}t \\&\quad = - \iint _{\Omega _{T-h}} \zeta _{\tau ,\varepsilon }' G([v_k]_h) \mathrm {d}x \mathrm {d}t \\&\quad \xrightarrow [h\downarrow 0]{}- \iint _{\Omega _T} \zeta _{\tau ,\varepsilon }' G(v_k) \mathrm {d}x \mathrm {d}t \\&\quad = \frac{1}{\varepsilon }\int ^{\tau +\varepsilon }_\tau \int _\Omega G(v_k)\mathrm {d}x \mathrm {d}t- \frac{1}{\varepsilon }\int ^{\varepsilon }_0 \int _\Omega G(v_k)\mathrm {d}x \mathrm {d}t, \\&\quad \xrightarrow [\varepsilon \downarrow 0]{}\int _\Omega G(v_k)(x,\tau )\mathrm {d}x. \end{aligned}$$

The limit of the second term vanishes since $G(v_k)(x,0)=G(\frac{1}{k}+\Psi (x))=0$ and $M\ge \sup _{\bar{\Omega }}\Psi +1$ by assumption. The limits exist since $\int _\Omega G(v_k)(x,t) \mathrm {d}x$ is continuous with respect to *t*. The continuity can be concluded from the fact that *G* is Lipschitz and $v_k\in C([0,T]; L^2(\Omega ))$. Therefore, we have after passing to the limits $h\downarrow 0$ and $\varepsilon \downarrow 0$ that

5.19 $$\begin{aligned} \int _\Omega&G(v_k)(x,\tau )\mathrm {d}x + \iint _{\Omega _\tau } A_k(v_k,\nabla v_k)\cdot \nabla \big (\tilde{v}_k^\beta -M^\beta \big )_+ \mathrm {d}x\mathrm {d}t \nonumber \\&= \iint _{\Omega _\tau } \big [f\big (\tilde{v}_k^\beta -M^\beta \big )_+ + k^{-\alpha }|\nabla z|^{p-2}\nabla z\cdot \nabla \big (\tilde{v}_k^\beta -M^\beta \big )_+\big ] \mathrm {d}x \mathrm {d}t \end{aligned}$$

holds true for any $\tau \in (0,T]$. Using the expression (5.3) for $A_k$, the fact that $\nabla (\tilde{v}_k^\beta -M^\beta )_+=0$ a.e. on the sets $\{(x,t)\in \Omega _T:v_k(x,t)\ge k\}$ and $\{(x,t)\in \Omega _T:v_k(x,t)\le M\}$, the chain rule and Lemma 3.5, we have

$$\begin{aligned}&A_k(v_k, \nabla v_k) \cdot \nabla \big (\tilde{v}_k^\beta -M^\beta \big )_+ \\&\quad = \tilde{v}_k^\alpha |\nabla v_k+\nabla z |^{p-2}(\nabla v_k+\nabla z) \cdot \nabla \big (\tilde{v}_k^\beta -M^\beta \big )_+ \\&\quad = \tilde{v}_k^\alpha |\nabla \tilde{v}_k+\nabla z |^{p-2}(\nabla \tilde{v}_k+\nabla z) \cdot \nabla \big (\tilde{v}_k^\beta -M^\beta \big )_+ \\&\quad = \beta ^{1-p} \chi _{\{\tilde{v}_k> M\}}|\nabla \tilde{v}_k^\beta +\beta \tilde{v}_k^{\beta -1}\nabla z|^{p-2}\big (\nabla \tilde{v}_k^\beta +\beta \tilde{v}_k^{\beta -1} \nabla z\big )\cdot \nabla \tilde{v}_k^\beta \\&\quad \ge \beta ^{1-p} \chi _{\{\tilde{v}_k> M\}} \big [2^{-p}|\nabla \tilde{v}_k^\beta |^p - 2^p\beta ^p \tilde{v}_k^{p(\beta -1)}|\nabla z|^p\big ] \\&\quad = 2^{-p}\beta ^{1-p} \big |\nabla (\tilde{v}_k^\beta -M^\beta )_+\big |^p -2^p\beta \chi _{\{\tilde{v}_k>M\}}\tilde{v}_k^{p(\beta -1)}|\nabla z|^p. \end{aligned}$$

Moreover, using the definition of *G* and Lemma 3.2 (i), we can estimate

$$\begin{aligned} G(v_k)\ge \chi _{\{\tilde{v}_k>M\}}\mathfrak {b}[\tilde{v},M]\ge c(\beta )\,\big (\tilde{v}_k^\frac{\beta +1}{2}-M^\frac{\beta +1}{2}\big )^2_+. \end{aligned}$$

The last term in the integral on the right-hand side of (5.19) can be estimated using the Schwarz inequality, Young’s inequality and the fact that $k>1$ as

$$\begin{aligned} k^{-\alpha }|\nabla z|^{p-2}\nabla z\cdot \nabla (\tilde{v}_k^\beta -M^\beta )_+&\le |\nabla z|^{p-1}|\nabla (\tilde{v}_k^\beta -M^\beta )_+| \\&\le \tfrac{\beta ^{1-p}}{2^{p+1}}|\nabla (\tilde{v}_k^\beta -M^\beta )_+|^p + c(\beta ,p)\, \chi _{\{\tilde{v}_k>M\}}|\nabla z|^p . \end{aligned}$$

The integral over the first term can be included in the first term on the left-hand side of (5.19), and we end up with

$$\begin{aligned}&\int _\Omega \big (\tilde{v}_k^\frac{\beta +1}{2}-M^\frac{\beta +1}{2}\big )^2_+(x,\tau )\mathrm {d}x + \iint _{\Omega _\tau } |\nabla (\tilde{v}_k^\beta -M^\beta )_+|^p\mathrm {d}x\mathrm {d}t\\&\qquad \le c \iint _{\Omega _\tau \cap \{\tilde{v}_k>M\}} \big [\tilde{v}_k^{p(\beta -1)} |\nabla z|^p + f(\tilde{v}_k^\beta -M^\beta )\big ] \mathrm {d}x \mathrm {d}t \end{aligned}$$

for any $\tau \in (0,T]$ and a constant $c=c(\beta ,p)$. Since $(\tilde{v}_k^\beta -M^\beta )_+\in L^p(0,T;W^{1,p}_0(\Omega ))$, we have by Young’s and Poincaré’s inequality for any $\varepsilon \in (0,1)$ that

$$\begin{aligned}&\iint _{\Omega _\tau \cap \{\tilde{v}_k>M\}} f(\tilde{v}_k^\beta -M^\beta ) \mathrm {d}x \mathrm {d}t\\&\qquad \le \varepsilon \iint _{\Omega _\tau } (\tilde{v}_k^\beta -M^\beta )_+^p \mathrm {d}x \mathrm {d}t + \varepsilon ^{-\frac{1}{p-1}}\iint _{\Omega _\tau \cap \{\tilde{v}_k>M\}} f^{p'} \mathrm {d}x \mathrm {d}t \\&\qquad \le \varepsilon c\iint _{\Omega _\tau } |\nabla (\tilde{v}_k^\beta -M^\beta )_+|^p \mathrm {d}x \mathrm {d}t + \varepsilon ^{-\frac{1}{p-1}}\iint _{\Omega _\tau \cap \{\tilde{v}_k>M\}} f^{p'} \mathrm {d}x \mathrm {d}t \end{aligned}$$

and

$$\begin{aligned}&\iint _{\Omega _\tau \cap \{\tilde{v}_k>M\}} \tilde{v}_k^{p(\beta -1)} |\nabla z|^p \mathrm {d}x \mathrm {d}t \\&\qquad \le \varepsilon \iint _{\Omega _\tau \cap \{\tilde{v}>M\}} \tilde{v}_k^{\beta p} \mathrm {d}x \mathrm {d}t + \varepsilon ^{-\frac{1}{\beta -1}}\iint _{\Omega _\tau \cap \{\tilde{v}_k>M\}} |\nabla z|^{\beta p} \mathrm {d}x \mathrm {d}t \\&\qquad \le \varepsilon c\iint _{\Omega _\tau } (\tilde{v}_k^\beta -M^\beta )_+^p \mathrm {d}x \mathrm {d}t + c\,\varepsilon ^{-\frac{1}{\beta -1}}\iint _{\Omega _\tau \cap \{\tilde{v}_k>M\}} \big [|\nabla z|^{\beta p} + M^{\beta p}\big ] \mathrm {d}x \mathrm {d}t\\&\qquad \le \varepsilon c\iint _{\Omega _\tau } |\nabla (\tilde{v}_k^\beta -M^\beta )_+|^p \mathrm {d}x \mathrm {d}t + c\,\varepsilon ^{-\frac{1}{\beta -1}}\iint _{\Omega _\tau \cap \{\tilde{v}_k>M\}} \big [|\nabla z|^{\beta p} + M^{\beta p}\big ] \mathrm {d}x \mathrm {d}t. \end{aligned}$$

Due to the Poincaré inequality, the constant *c* depends on $\Omega$. Choosing $\varepsilon$ small enough, we can reabsorb the terms involving $|\nabla (\tilde{v}^\beta -M^\beta )_+|^p$ into the left-hand side. This leads us to

$$\begin{aligned}&\int _\Omega \big (\tilde{v}_k^\frac{\beta +1}{2}-M^\frac{\beta +1}{2}\big )^2_+(x,\tau )\mathrm {d}x + \iint _{\Omega _\tau } |\nabla (\tilde{v}_k^\beta -M^\beta )_+|^p\mathrm {d}x\mathrm {d}t \\&\qquad \le c \iint _{\Omega _\tau \cap \{\tilde{v}_k>M\}} \big [|\nabla z|^{\beta p} + f^{p'} + M^{\beta p}\big ] \mathrm {d}x \mathrm {d}t. \end{aligned}$$

In the first term on the right-hand side, we take the supremum over $\tau \in [0,T]$, while in the second one we choose $\tau =T$. Proceeding in this way, we end up with inequality (5.18). $\square$

We utilize the previous lemma to show that the integral of $\tilde{v}_k^{\beta p}$ is bounded independently of *k*.

#### Corollary 5.5

Let $k>1$ and $v_k$ be an admissible weak solution to the Cauchy–Dirichlet problem (5.7) in the sense of Definition 5.1. Then, there is a constant *c* depending only on $\beta$, *p* and the domain $\Omega$ such that the function $\tilde{v}_k$ defined in (5.17) satisfies

$$\begin{aligned} \iint _{\Omega _T}\tilde{v}_k^{\beta p}\mathrm {d}x \mathrm {d}t \le c\,K, \end{aligned}$$

where

5.20 $$\begin{aligned} K := \iint _{\Omega _T } \big [|\nabla z|^{\beta p}+ f^{p'}\big ] \mathrm {d}x\mathrm {d}t + \Big (\sup _{\bar{\Omega }}\Psi ^{\beta p} + 1\Big ) |\Omega _T|. \end{aligned}$$

#### Proof

We define $M_o=\sup _{\bar{\Omega }}\Psi +1$ and observe that $(\tilde{v}_k^\beta -M_o^\beta )_+\in L^p(0,T;W^{1,p}_0(\Omega ))$. Combining Poincaré’s inequality applied slice-wise for a.e. $t\in (0,T)$ and the energy estimate from Lemma 5.4, we obtain

$$\begin{aligned} \iint _{\Omega _T} (\tilde{v}_k^\beta -M_o^\beta )_+^p\mathrm {d}x\mathrm {d}t&\le c\iint _{\Omega _T}|\nabla (\tilde{v}_k^\beta -M_o^\beta )_+|^p\mathrm {d}x \mathrm {d}t\\&\le c\iint _{\Omega _T} \big [|\nabla z|^{\beta p} + f^{p'} + M_o^{\beta p}\big ]\mathrm {d}x\mathrm {d}t. \end{aligned}$$

Thus, we have

$$\begin{aligned} \iint _{\Omega _T} \tilde{v}_k^{\beta p} \mathrm {d}x\mathrm {d}t&\le c \iint _{\Omega _T} (\tilde{v}_k^\beta -M_o^\beta )_+^p\mathrm {d}x\mathrm {d}t + c\,M_o^{\beta p}|\Omega _T| \\&\le c \iint _{\Omega _T} \big [|\nabla z|^{\beta p} + f^{p'}\big ]\mathrm {d}x\mathrm {d}t + c \, M_o^{\beta p} | \Omega _T|, \end{aligned}$$

with a constant $c=c(\beta ,p,\Omega )$. This is the desired bound for the $L^{\beta p}$-norm of $\tilde{v}_k$. $\square$

Now, we are ready to prove the boundedness result.

#### Lemma 5.6

Let $k>1$ and $v_k$ be an admissible weak solution to the Cauchy–Dirichlet problem (5.7) in the sense of Definition 5.1. Then, there is a constant $L>0$ depending only on $n,\beta , p, \Omega _T, f, \psi , z$, and $\sigma$ (and thus independent of *k*) such that for every $k> L$ we have

$$\begin{aligned} v_k\le L \quad \text{ a.e. } \text{ in } \Omega _T. \end{aligned}$$

#### Proof

For $M\ge \sup _{\bar{\Omega }}\Psi +1$, we define the sequences

$$\begin{aligned} M_j:=M(2-2^{-j})^\frac{2}{\beta +1},\quad Y_j:=\iint _{\Omega _T}\big (\tilde{v}_k^\frac{\beta +1}{2}-M^\frac{\beta +1}{2}_j \big )_+^\frac{2\beta p}{\beta +1}\mathrm {d}x\mathrm {d}t, j\in \mathbb {N}_0, \end{aligned}$$

where $\tilde{v}_k$ is defined in (5.17). Furthermore, we denote $m:=\frac{\beta +1}{\beta }$ and $A_j:=\Omega _T \cap \{ \tilde{v}_k>M_j\}$. Note that

$$\begin{aligned} \nabla \big (\tilde{v}_k^\beta - M_{j+1}^\beta \big )_+&= \chi _{\{\tilde{v}_k>M_{j+1}\}}\nabla \tilde{v}_k^\beta =\chi _{\{\tilde{v}_k>M_{j+1}\}}\nabla (\tilde{v}_k^\frac{\beta +1}{2} )^\frac{2\beta }{\beta +1} \\&=\tfrac{2\beta }{\beta +1}\chi _{\{\tilde{v}_k>M_{j+1}\}}(\tilde{v}_k^\frac{\beta +1}{2})^\frac{\beta -1}{\beta +1}\nabla \tilde{v}_k^\frac{\beta +1}{2} = \tfrac{2\beta }{\beta +1}\tilde{v}_k^\frac{\beta -1}{2}\nabla \big (\tilde{v}_k^\frac{\beta +1}{2}-M_{j+1}^\frac{\beta +1}{2}\big )_+. \end{aligned}$$

Thus,

5.21 $$\begin{aligned} \big |\nabla \big (\tilde{v}_k^\beta - M_{j+1}^\beta \big )_+\big |&= \tfrac{2\beta }{\beta +1}\tilde{v}_k^\frac{\beta -1}{2}\big |\nabla \big (\tilde{v}_k^\frac{\beta +1}{2}-M_{j+1}^\frac{\beta +1}{2}\big )_+\big | \nonumber \\&\ge \tfrac{2\beta }{\beta +1}\big (\tilde{v}_k^\frac{\beta +1}{2}-M_{j+1}^\frac{\beta +1}{2}\big )_+^{\frac{2\beta }{\beta +1}-1}\big |\nabla \big (\tilde{v}_k^\frac{\beta +1}{2}-M_{j+1}^\frac{\beta +1}{2}\big )_+\big | \nonumber \\&=\big |\nabla \big (\tilde{v}_k^\frac{\beta +1}{2}-M_{j+1}^\frac{\beta +1}{2}\big )_+^\frac{2\beta }{\beta +1}\big |. \end{aligned}$$

Using Hölder’s inequality, Gagliardo Nirenberg’s inequality from Lemma 3.8, (5.21) and the energy estimate from Lemma 5.4, we infer that

$$\begin{aligned}&Y_{j+1} \le \bigg [ \iint _{\Omega _T}\Big [\big (\tilde{v}_k^\frac{\beta +1}{2}-M^\frac{\beta +1}{2}_{j+1}\big )_+^\frac{2\beta }{\beta +1} \Big ]^{p\frac{n+m}{n}}\mathrm {d}x\mathrm {d}t \bigg ]^\frac{n}{n+m}|A_{j+1}|^\frac{m}{n+m} \\&\quad \le c \bigg [\sup _{\tau \in [0,T]}\int _\Omega \big (\tilde{v}_k^\frac{\beta +1}{2}-M^\frac{\beta +1}{2}_{j+1}\big )_+^2(\tau )\mathrm {d}x\bigg ]^\frac{p}{n+m} \\&\quad \cdot \bigg [ \iint _{\Omega _T} \Big |\nabla \big (\tilde{v}_k^\frac{\beta +1}{2}-M^\frac{\beta +1}{2}_{j+1}\big )_+^\frac{2\beta }{\beta +1} \Big |^p \mathrm {d}x \mathrm {d}t \bigg ]^\frac{n}{n+m}|A_{j+1}|^\frac{m}{n+m} \\&\quad = c \bigg [\sup _{\tau \in [0,T]}\int _\Omega \big (\tilde{v}_k^\frac{\beta +1}{2}-M^\frac{\beta +1}{2}_{j+1}\big )_+^2(\tau )\mathrm {d}x\bigg ]^\frac{p}{n+m} \\&\quad \bigg [ \iint _{\Omega _T} \big |\nabla \big (\tilde{v}_k^\beta -M^\beta _{j+1}\big )_+\big |^p \mathrm {d}x \mathrm {d}t \bigg ]^\frac{n}{n+m}|A_{j+1}|^\frac{m}{n+m} \\&\quad \le c\bigg [ \iint _{A_{j+1}} \big [|\nabla z|^{\beta p}+ f^{p'} + M_{j+1}^{\beta p}\big ]\mathrm {d}x\mathrm {d}t \bigg ]^\frac{n+p}{n+m}|A_{j+1}|^\frac{m}{n+m}. \end{aligned}$$

With the abbreviation $G:=|\nabla z|^{\beta p}+ f^{p'}$, we obtain

5.22 $$\begin{aligned} Y_{j+1}&\le c\bigg [\iint _{A_{j+1}} G \mathrm {d}x\mathrm {d}t + M_{j+1}^{\beta p}|A_{j+1}|\bigg ]^\frac{n+p}{n+m}|A_{j+1}|^\frac{m}{n+m} \nonumber \\&\le c\Big [\Vert G\Vert _{L^\sigma (\Omega _T)}|A_{j+1}|^{1-\frac{1}{\sigma }} + M_{j+1}^{\beta p}|A_{j+1}|\Big ]^\frac{n+p}{n+m}|A_{j+1}|^\frac{m}{n+m}. \end{aligned}$$

We can estimate the measure of $A_{j+1}$ by noting that

$$\begin{aligned} |A_{j+1}|&=M^{-\beta p} 2^{(j+1)\frac{2 \beta p}{\beta +1}}|A_{j+1}|\big (M^\frac{\beta +1}{2}_{j+1}-M^\frac{\beta +1}{2}_j\big )^\frac{2\beta p}{\beta + 1} \\&\le M^{-\beta p} 2^{(j+1)\frac{2 \beta p}{\beta +1}}\iint _{A_{j+1}}\big (\tilde{v}_k^\frac{\beta +1}{2}-M^\frac{\beta +1}{2}_j\big )_+^\frac{2\beta p}{\beta + 1}\mathrm {d}x \mathrm {d}t \\&\le M^{-\beta p} 2^{(j+1)\frac{2 \beta p}{\beta +1}}Y_j. \end{aligned}$$

We use this to estimate the second term in the square brackets of (5.22) and the last factor. The remaining instance of $|A_{j+1}|$ is treated in the same way except that we drop the factor containing *M*, which is possible since $M>1$. In this way, we obtain

$$\begin{aligned} Y_{j+1}&\le c\Big [\Vert G\Vert _{L^\sigma }2^{j(1-\frac{1}{\sigma })\frac{2 \beta p}{\beta +1}}Y_j^{1-\frac{1}{\sigma }} + 2^{j\frac{2 \beta p}{\beta +1}}Y_j \Big ]^\frac{n+p}{n+m}(M^{-\beta p} 2^{j\frac{2 \beta p}{\beta +1}}Y_j)^\frac{m}{n+m}\\&\le c\, M^{-\frac{\beta pm}{n+m}} \Big [\Vert G\Vert _{L^\sigma (\Omega _T)} + Y_j^\frac{1}{\sigma } \Big ]^\frac{n+p}{n+m} 2^{j\frac{2 \beta p}{\beta +1} \frac{n+m+p}{n+m}}Y_j^{(1-\frac{1}{\sigma })\frac{n+p}{n+m}+\frac{m}{n+m}} \\&\le c\, M^{-\frac{\beta pm}{n+m}}\Big [\Vert G\Vert _{L^\sigma (\Omega _T)} + \Vert \tilde{v}_k\Vert _{L^{\beta p}(\Omega _T)}^\frac{\beta p}{\sigma } \Big ]^\frac{n+p}{n+m} 2^{j\frac{2 \beta p}{\beta +1} \frac{n+m+p}{n+m}}Y_j^{1 +\frac{1}{n+m}(p-\frac{1}{\sigma }(n+p))}. \end{aligned}$$

In view of Corollary 5.5, this shows that

$$\begin{aligned} Y_{j+1} \le c\, M^{-\frac{\beta pm}{n+m}}\Big [\Vert G\Vert _{L^\sigma (\Omega _T)} + K^\frac{1}{\sigma } \Big ]^\frac{n+p}{n+m} 2^{j\frac{2 \beta p}{\beta +1} \frac{n+m+p}{n+m}}Y_j^{1 +\frac{1}{n+m}(p-\frac{1}{\sigma }(n+p))}, \end{aligned}$$

where *K* is defined in (5.20). Thus, we have verified the iterative estimate of Lemma 3.7 with the choices

$$\begin{aligned} C&:=c\, M^{-\frac{\beta pm}{n+m}} \Big [\Vert G\Vert _{L^\sigma (\Omega _T)} + K^\frac{1}{\sigma } \Big ]^\frac{n+p}{n+m},\;b:=2^{\frac{2 \beta p}{\beta +1} \frac{n+m+p}{n+m}} ,\;\\ \delta&:=\tfrac{1}{n+m}(p-\tfrac{1}{\sigma }(n+p)). \end{aligned}$$

Note that $\delta > 0$ since $\sigma >\frac{n+p}{p}$. In order to apply Lemma 3.7, we also need to have

$$\begin{aligned} Y_0\le C^{-\frac{1}{\delta }}b^{-\frac{1}{\delta ^2}}. \end{aligned}$$

Since $Y_0\le \Vert \tilde{v}_k\Vert _{L^{\beta p}(\Omega _T)}^{\beta p}\le c\,K$, it is sufficient that

$$\begin{aligned} c\, K\le C^{-\frac{1}{\delta }}b^{-\frac{1}{\delta ^2}}, \end{aligned}$$

which, using the definitions of *C* and *b*, is equivalent to

$$\begin{aligned} M\ge \tilde{c}\,K^\frac{\delta (n+m)}{\beta p m} \Big [\Vert G\Vert _{L^\sigma (\Omega _T)} + K^\frac{1}{\sigma } \Big ]^\frac{n+p}{\beta p m} b^{\frac{n+m}{\delta \beta pm}}. \end{aligned}$$

for a constant $\tilde{c}$ depending on $n, p, \beta , \Omega$. We now choose

$$\begin{aligned} M:= \max \bigg \{\sup _{\bar{\Omega }}\Psi +1, \tilde{c}\,K^\frac{\delta (n+m)}{\beta p m} \Big [\Vert G\Vert _{L^\sigma (\Omega _T)} + K^\frac{1}{\sigma } \Big ]^\frac{n+p}{\beta p m} b^{\frac{n+m}{\delta \beta pm}} \bigg \}. \end{aligned}$$

Thus, *M* is a constant depending only on $n, p, \beta , \Omega _T, f, z, \sigma$. Since $Y_j\rightarrow 0$ with this choice, we have that

$$\begin{aligned} \Vert T_k\circ v_k\Vert _{L^\infty (\Omega _T)}=\Vert \tilde{v}_k\Vert _{L^\infty (\Omega _T)}\le 2M =: L, \end{aligned}$$

for all *k*. But this means that for $k>L$, we have

$$\begin{aligned} \Vert v_k\Vert _{L^\infty (\Omega _T)}\le L, \end{aligned}$$

which proves the claim. $\square$

The previous lemma together with Lemma 5.3 and the definition of $A_k$ show that

$$\begin{aligned} A_k(v_k,\nabla v_k)=A\big (v_k,\nabla v_k^\beta \big ), \end{aligned}$$

for large *k*. Thus, for large *k*, we deduce from the differential equation (5.8) that $v_k$ satisfies also the equation

5.23 $$\begin{aligned} \iint _{\Omega _T}\big [A\big (v_k,\nabla v_k^\beta \big )\cdot \nabla \varphi - v_k\partial _t\varphi \big ] \mathrm {d}x\mathrm {d}t = \iint _{\Omega _T} \big [f\varphi + k^{-\alpha }|\nabla z|^{p-2}\nabla z\cdot \nabla \varphi \big ] \mathrm {d}x \mathrm {d}t, \end{aligned}$$

for any $\varphi \in C_0^\infty (\Omega _T)$. We now show a generalization of this result for test functions which do not necessarily vanish at time zero. This result will be used to verify that once we have concluded the existence of a solution to the original equation, it will also satisfy the correct boundary value.

#### Lemma 5.7

For sufficiently large *k* and $\varphi \in C^\infty (\bar{\Omega }\times [0,T])$ with support in $K\times [0,\tau ]$ where $K\subset \Omega$ is compact and $\tau \in (0,T)$, we have

5.24 $$\begin{aligned} \iint _{\Omega _T}\big [A\big (v_k,\nabla v_k^\beta \big )\cdot \nabla \varphi - v_k\partial _t\varphi \big ] \mathrm {d}x\mathrm {d}t&= \iint _{\Omega _T} \big [f\varphi + k^{-\alpha }|\nabla z|^{p-2}\nabla z\cdot \nabla \varphi \big ] \mathrm {d}x \mathrm {d}t \nonumber \\&\quad + \int _\Omega v_k(0)\varphi (0)\mathrm {d}x. \end{aligned}$$

#### Proof

For $\varphi$ as in the statement of the lemma, we apply (5.23) with the test function $\zeta _\varepsilon \varphi$, where

$$\begin{aligned} \zeta _\varepsilon (t)= {\left\{ \begin{array}{ll} \frac{1}{\varepsilon }t, &{}t\in [0,\varepsilon ], \\ 1, &{}t>\varepsilon , \end{array}\right. } \end{aligned}$$

and pass to the limit $\varepsilon \downarrow 0$. This is possible since $v_k \in C([0,T];L^2(\Omega ))$. $\square$

Similarly as in Lemma 3.10, we can show that the weak solution $v_k$ also satisfies the following modified weak form.

#### Lemma 5.8

Let $k>1$ and $v_k$ be an admissible weak solution to the Cauchy–Dirichlet problem (5.7) in the sense of Definition 5.1. Then, there holds

5.25 $$\begin{aligned} \iint _{\Omega _T} \zeta ' \mathfrak {b}[v_k,w] \mathrm {d}x\mathrm {d}t&= \iint _{\Omega _T} \zeta \Big [\partial _t w^\beta (v_k-w) + A(v_k,\nabla v_k^\beta )\cdot \big (\nabla v_k^\beta -\nabla w^\beta \big ) \Big ] \mathrm {d}x\mathrm {d}t \nonumber \\&\quad - \iint _{\Omega _T} \zeta \Big [ f(v_k^\beta -w^\beta ) + k^{-\alpha } |\nabla z|^{p-2}\nabla z \cdot \big (\nabla v_k^\beta -\nabla w^\beta \big )\Big ] \mathrm {d}x\mathrm {d}t , \end{aligned}$$

for all $w^\beta \in k^{-\beta } + L^p(0,T;W_0^{1,p}(\Omega ))$ with $\partial _t w^\beta \in L^{\frac{\beta +1}{\beta }}(\Omega _T)$ and any $\zeta \in W^{1,\infty }([0,T],\mathbb {R}_{\ge 0})$ with $\zeta (0)=0=\zeta (T)$.

The next step is a uniform $L^p$-bound for the gradients of the functions $v_k^\beta$.

#### Lemma 5.9

Let $k>1$ and $v_k$ be an admissible weak solution to the Cauchy–Dirichlet problem (5.7) in the sense of Definition 5.1. Then, there is a constant $C>0$ depending on $n,\beta , p, \Omega _T, f, \psi$, and *z* such that

$$\begin{aligned} \iint _{\Omega _T}|\nabla v_k^\beta |^p\mathrm {d}x \mathrm {d}t \le C, \end{aligned}$$

for all $$k\in \mathbb {N}$$.

#### Proof

For $\delta \in (0,\frac{T}{2})$, we define

$$\begin{aligned} \zeta _\delta (t):= {\left\{ \begin{array}{ll}\frac{1}{\delta }t, \quad &{}t\in [0,\delta ],\\ 1, &{}t\in [\delta ,T-\delta ], \\ \frac{1}{\delta }(T-t), &{}t\in [T-\delta ,T]. \end{array}\right. } \end{aligned}$$

and choose $\zeta =\zeta _\delta$ and the comparison function $w=\frac{1}{k}$ in the modified weak form (5.25) of the differential equation. Since $\partial _t w=0$, the first term on the right-hand side is zero. Our goal now is to pass to the limit $\delta \downarrow 0$. Note that $\nabla w = 0$ and thus

$$\begin{aligned} \iint _{\Omega _T} \zeta _\delta A(v_k,\nabla v_k^\beta )\cdot \big (\nabla v_k^\beta -\nabla w^\beta \big ) \mathrm {d}x \mathrm {d}t&\xrightarrow [\delta \downarrow 0]{} \iint _{\Omega _T} A\big (v_k,\nabla v_k^\beta \big )\cdot \nabla v_k^\beta \mathrm {d}x \mathrm {d}t. \end{aligned}$$

Using the definition of the vector field *A* in (2.5), Lemmas 3.5 and 5.6, we obtain

$$\begin{aligned} A\big (v_k,\nabla v_k^\beta \big )\cdot \nabla v_k^\beta&\ge \beta ^{1-p}\big [2^{-p} |\nabla v_k^\beta |^p -2^p \beta ^p v_k^{p(\beta -1)}|\nabla z|^p\big ] \\&\ge 2^{-p}\beta ^{1-p} |\nabla v_k^\beta |^p -2^p\beta L^{p(\beta -1)}|\nabla z|^p. \end{aligned}$$

For the term on the left-hand side, we have

$$\begin{aligned} \iint _{\Omega _T} \zeta _\delta ' \mathfrak {b}[v_k,w] \mathrm {d}x\mathrm {d}t&= \frac{1}{\delta } \int _0^\delta \int _{\Omega } \mathfrak {b}[v_k,\tfrac{1}{k}] \mathrm {d}x\mathrm {d}t - \frac{1}{\delta } \int _{T-\delta }^T\int _{\Omega } \mathfrak {b}[v_k,\tfrac{1}{k}] \mathrm {d}x\mathrm {d}t \\&\le \frac{1}{\delta } \int _0^\delta \int _{\Omega } \mathfrak {b}[v_k,\tfrac{1}{k}] \mathrm {d}x\mathrm {d}t \\&\xrightarrow [\delta \downarrow 0]{} \int _{\Omega } \mathfrak {b}[\tfrac{1}{k}+\Psi ,\tfrac{1}{k}] \mathrm {d}x\mathrm {d}t \\&\le \frac{1}{\beta +1}\int _\Omega (\Psi +1)^{\beta +1}\mathrm {d}x . \end{aligned}$$

We were able to omit the integral over $[T-\delta ,T]$ since $\mathfrak {b}[v,\tfrac{1}{k}]$ is always nonnegative. Passing to the limits in the remaining terms on the right-hand side presents no problems, and taking into account the previous estimates we end up with

$$\begin{aligned} \iint _{\Omega _T}|\nabla v_k^\beta |^p\mathrm {d}x \mathrm {d}t&\le c\,T L^{p(\beta -1)}\int _{\Omega }|\nabla z|^p\mathrm {d}x + c \int _\Omega (\Psi +1)^{\beta +1}\mathrm {d}x\\&\quad + c\,L^\beta \iint _{\Omega _T} |f| \mathrm {d}x\mathrm {d}t + c\iint _{\Omega _T}|\nabla z|^{p-1} |\nabla v_k^\beta |\mathrm {d}x \mathrm {d}t, \end{aligned}$$

where $c=c(p,\beta )$. Using Young’s inequality in the last integral, we finally obtain

$$\begin{aligned} \iint _{\Omega _T}|\nabla v_k^\beta |^p\mathrm {d}x \mathrm {d}t&\le c\bigg [\int _\Omega \big [L^{p(\beta -1)}|\nabla z|^p + (\Psi +1)^{\beta +1}\big ]\mathrm {d}x + L^\beta \iint _{\Omega _T}|f| \mathrm {d}x \mathrm {d}t \bigg ], \end{aligned}$$

for a constant *c* depending on $\beta$ and *p*. $\square$

## Proof of the main result

Lemmas 5.6 and 5.9 show that $(v_k^\beta )$ is a bounded sequence in the reflexive Banach space $L^p(0,T;W^{1,p}(\Omega ))$. Therefore, there is a subsequence converging weakly to an element of $L^p(0,T;W^{1,p}(\Omega ))$. This element can be regarded as a nonnegative function on $\Omega _T$ since every $v_k^\beta$ is nonnegative, and hence we can write the limit as $v^\beta$ for some nonnegative function *v*. (Mazur’s lemma provides us with a subsequence of nonnegative functions converging in $L^p(\Omega _T)$ and from this we obtain yet another subsequence converging pointwise a.e. in $\Omega _T$.) We even have a stronger form of convergence.

### Lemma 6.1

Let $(v_k)$ be the weak solutions to the Cauchy–Dirichlet problems (5.7) in the sense of Definition 5.1. Then, there exists a subsequence $(k_j)_{j\in \mathbb {N}}$ with $k_j\rightarrow \infty$ as $j\rightarrow \infty$ and a nonnegative function $v\in L^{\infty }(\Omega _T)$ with $v^\beta \in L^p(0,T;W^{1,p}_0(\Omega ))$ such that

$$\begin{aligned} \left\{ \begin{array}{ll} v_{k_j}^\beta \rightharpoondown v^\beta &{}\quad \text{ weakly } \text{ in } L^p(0,T;W^{1,p}(\Omega )), \\ v_{k_j}\rightarrow v &{}\quad \text{ strongly } \text{ in } L^{q}(\Omega _T) \text{ for } \text{ any } q\ge 1 \text{ and } \text{ a.e. } \text{ in } \Omega _T. \end{array} \right. \end{aligned}$$

### Proof

From Lemma 5.9, we know that $(v_k^\beta -k^{-\beta })$ is a bounded sequence in the reflexive Banach space $L^p(0,T;W_0^{1,p}(\Omega ))$. Therefore, there is a subsequence $(k_j)_{j\in \mathbb {N}}$ with $k_j\rightarrow \infty$ as $j\rightarrow \infty$ and a function $w\in L^p(0,T;W^{1,p}_0(\Omega ))$ such that $v_{k_j}^\beta -k^{-\beta }\rightharpoondown w$ weakly in $L^p(0,T;W^{1,p}(\Omega ))$. This implies that also

6.1 $$\begin{aligned} v_{k_j}^\beta \rightharpoondown w\quad \text{ weakly } \text{ in } L^p(0,T;W^{1,p}(\Omega ))\text{. } \end{aligned}$$

Our next aim is to ensure strong convergence of $(v_{k_j})$ and thereby to identify the limit function as the pointwise a.e. limit of the subsequence. For this purpose, we let $\tau \in (0,T)$. For $h\in (0,T-\tau )$ and $\delta \in (0,\min \{\tau ,T-\tau -h\})$, we define

$$\begin{aligned} \zeta _\delta (t):={\left\{ \begin{array}{ll} 0, &{}t<\tau -\delta \\ \frac{1}{\delta }(t-\tau +\delta ), &{}t\in [\tau -\delta ,\tau ] \\ 1, &{}t \in (\tau ,\tau +h) \\ \frac{1}{\delta }(\tau +h+\delta -t), &{}t\in [\tau +h,\tau +h+\delta ] \\ 0, &{}t> \tau +h+\delta \end{array}\right. } \end{aligned}$$

and consider $\varphi \in C^\infty _0(\Omega )$. For $k>1$, we use the weak formulation (5.23) with the test function $\zeta _\delta \varphi$ and obtain

$$\begin{aligned} \frac{1}{\delta }\int ^\tau _{\tau -\delta }&\int _\Omega v_k \varphi \mathrm {d}x \mathrm {d}t - \frac{1}{\delta }\int ^{\tau +h+\delta }_{\tau +h}\int _\Omega v_k \varphi \mathrm {d}x \mathrm {d}t \\&= \iint _{\Omega _T} \big [\zeta _\delta A\big (v_k,\nabla v_k^\beta \big )\cdot \nabla \varphi -\zeta _\delta f\varphi -\zeta _\delta k^{-\alpha }|\nabla z|^{p-2}\nabla z \cdot \nabla \varphi \big ]\mathrm {d}x \mathrm {d}t. \end{aligned}$$

Passing to the limit $\delta \rightarrow 0$, we see that for any $\tau \in (0,T)$ and $h\in (0,T-\tau )$ there holds

$$\begin{aligned}&\int _\Omega [v_k(\tau )-v_k(\tau +h)]\varphi \mathrm {d}x \\&\quad = \int ^{\tau +h}_\tau \int _\Omega \big [A\big (v_k,\nabla v_k^\beta \big )\cdot \nabla \varphi -f\varphi -k^{-\alpha }|\nabla z|^{p-2}\nabla z \cdot \nabla \varphi \big ] \mathrm {d}x \mathrm {d}t. \end{aligned}$$

Regarding $v_k$ for fixed times as an element of $(W^{1,p}_0(\Omega ))'$, and letting $\langle \cdot , \cdot \rangle$ denote the dual pairing of $(W^{1,p}_0(\Omega ))'$ and $W^{1,p}_0(\Omega )$, we thus have

$$\begin{aligned} |\langle v_k(\tau )-v_k(\tau +h),\varphi \rangle |&\le \int ^{\tau +h}_\tau \int _\Omega \Big [\big [|A(v_k,\nabla v_k^\beta )|+|\nabla z|^{p-1}\big ]|\nabla \varphi | + |f||\varphi |\Big ]\mathrm {d}x \mathrm {d}t\\&\le c\int ^{\tau +h}_\tau \int _\Omega \Big [\big [|\nabla v_k|^{p-1}+L^{\alpha }|\nabla z|^{p-1}\big ]|\nabla \varphi | + |f||\varphi |\Big ]\mathrm {d}x \mathrm {d}t, \end{aligned}$$

where in the last line we used the bound for $v_k$ from Lemma 5.6. By Hölder’s inequality, we continue to estimate

$$\begin{aligned} |\langle v_k(\tau )-v_k(\tau +h), \varphi \rangle |&\le c\int ^{\tau +h}_\tau \bigg [\int _\Omega \big [|\nabla v_k^\beta |^p+|\nabla z|^p\big ]\mathrm {d}x \bigg ]^\frac{p-1}{p}\Vert \nabla \varphi \Vert _{L^p(\Omega )} \mathrm {d}t \\&\quad + c\int ^{\tau +h}_\tau \bigg [\int _\Omega |f|^\frac{p}{p-1}\mathrm {d}x\bigg ]^\frac{p-1}{p}\Vert \varphi \Vert _{L^p(\Omega )}\mathrm {d}t \\&\le c\, h^\frac{1}{p} \Vert \varphi \Vert _{W^{1,p}(\Omega )}\bigg [\iint _{\Omega _T} \big [|\nabla v_k^\beta |^p+|\nabla z|^p + |f|^\frac{p}{p-1}\big ]\mathrm {d}x \mathrm {d}t\bigg ]^\frac{p-1}{p}, \end{aligned}$$

where $c=c(\alpha ,p,L)$. In light of Lemma 5.9, the expression in the square brackets is bounded by a constant independent of *k*. Thus, by the density of $C^\infty _0(\Omega )$ in $W^{1,p}_0(\Omega )$, we have shown that for almost all $\tau \in (0,T)$ and $h\in (0,T-\tau )$ there holds

$$\begin{aligned} \Vert v_k(\tau )-v_k(\tau +h)\Vert _{(W^{1,p}_0(\Omega ))'}\le c\, h^\frac{1}{p}, \end{aligned}$$

for a constant *c* independent of *k*. Moreover, we recall from Lemmas 5.6 and 5.9 that $v_k$ is a bounded sequence in $L^\infty (\Omega _T)$ and $v_k^\beta$ in $L^p(0,T;W^{1,p}(\Omega ))$. We have thus ensured that the assumptions of Corollary 4.6 (i) are satisfied with $Y=(W^{1,p}_0(\Omega ))'$, $m=\beta$ and $q=\mu =p$. Therefore, the application of Corollary 4.6 (i) to $(v_{k_j})$ ensures that $(v^\beta _{k_j})$ is relatively compact in $L^{p}(\Omega _T)$. In particular, there exists a strongly convergent subsequence and by virtue of (6.1) this implies the strong convergence of the whole sequence $(v^\beta _{k_j})$ to the limit function *w*, i.e., we have that

6.2 $$\begin{aligned} v_{k_j}^\beta \rightarrow w\quad \text{ strongly } \text{ in } L^p(\Omega _T)\text{. } \end{aligned}$$

By passing to another subsequence, we also obtain that $v_k^\beta$ converges to *w* pointwise a.e. in $\Omega _T$ and the uniform boundedness of $v_k$ ensures that also $w\in L^\infty (\Omega _T)$. We now define $v\in L^\infty (\Omega _T)$ via $v^\beta =w$, so that $v_k^\beta \rightharpoondown v^\beta$ weakly in $L^p(0,T;W^{1,p}(\Omega ))$ and $v_k^\beta \rightarrow v^\beta$ strongly in $L^p(\Omega _T)$ by (6.1) and (6.2). The uniform boundedness of the sequence $v_k$ together with the strong $L^p$-convergence $v_k^\beta \rightarrow v^\beta$ implies that $v_k\rightarrow v$ strongly in $L^q(\Omega _T)$ for any $q\ge 1$. $\square$

The next step of our argument is to show the strong convergence of the gradients.

### Lemma 6.2

Let the assumptions of Lemma 6.1 be in force. Then, for the subsequence $(k_j)_{j\in \mathbb {N}}$ there additionally holds that

$$\begin{aligned} \nabla v_{k_j}^\beta \rightarrow \nabla v^\beta \quad \text{ strongly } \text{ in } L^p(\Omega \times I) \end{aligned}$$

for any closed subinterval *I* of (0, *T*).

### Proof

Due to Remark 3.4, we know that for any $k>1$ there holds

$$\begin{aligned}{}[A(v_k,\nabla v_k^\beta )&-A(v_k,\nabla v^\beta )] \cdot (\nabla v_k^\beta - \nabla v^\beta ) \ge {\left\{ \begin{array}{ll} c\,|\nabla v_k^\beta -\nabla v^\beta |^p, &{}p\ge 2 \\ c\, V_k^{p-2}|\nabla v_k^\beta -\nabla v^\beta |^2, &{} p<2, \end{array}\right. } \end{aligned}$$

for a constant $c=c(p)$ and where

$$\begin{aligned} V_k:=\big (|\nabla v_k^\beta +\beta v_k^{\beta -1}\nabla z|^2 + |\nabla v^\beta +\beta v_k^{\beta -1}\nabla z|^2\big )^\frac{1}{2}. \end{aligned}$$

The expression $V_k^{p-2}$ which appears in the case $p<2$ is not defined if $V_k=0$, but this can occur only if $\nabla v^\beta =\nabla v_k^\beta$ and will therefore cause no problems. Now, let $\zeta \in C^\infty _0((0,T);[0,1])$. When $p<2$, Hölder’s inequality implies that

$$\begin{aligned} \iint _{\Omega _T}&\zeta |\nabla v_k^\beta -\nabla v^\beta |^p\mathrm {d}x \mathrm {d}t \\&\le \iint _{\Omega _T \cap \{ \nabla v_k^\beta \ne \nabla v^\beta \} } \zeta |\nabla v_k^\beta -\nabla v^\beta |^p V_k^{\frac{p}{2}(p-2)} V_k^{\frac{p}{2}(2-p)}\mathrm {d}x \mathrm {d}t \\&\le \bigg [\iint _{\Omega _T \cap \{ \nabla v_k^\beta \ne \nabla v^\beta \} } \zeta V_k^{p-2}|\nabla v_k^\beta -\nabla v^\beta |^2\mathrm {d}x \mathrm {d}t\bigg ]^\frac{p}{2}\bigg [\iint _{\Omega _T}\zeta V_k^p\mathrm {d}x \mathrm {d}t\bigg ]^\frac{2-p}{2} \\&\le c\bigg [\iint _{\Omega _T} \zeta [A(v_k,\nabla v_k^\beta )-A(v_k,\nabla v^\beta )] \cdot (\nabla v_k^\beta - \nabla v^\beta )\mathrm {d}x \mathrm {d}t\bigg ]^\frac{p}{2} \\&\quad \cdot \bigg [\iint _{\Omega _T}|\nabla v_k^\beta |^p + |\nabla v^\beta |^p + v_k^{p(\beta -1)}|\nabla z|^p\mathrm {d}x \mathrm {d}t \bigg ]^\frac{2-p}{2}. \end{aligned}$$

By Lemmas 5.6 and 5.9, the expression on the last row is bounded by a constant independent of *k*. The previous observations show that regardless of the value of *p*,

6.3 $$\begin{aligned} \frac{1}{c}\bigg [ \iint _{\Omega _T}&\zeta |\nabla v_k^\beta -\nabla v^\beta |^p\mathrm {d}x \mathrm {d}t \bigg ]^\nu \nonumber \\&\le \iint _{\Omega _T} \zeta [A(v_k,\nabla v_k^\beta )-A(v_k,\nabla v^\beta )] \cdot (\nabla v_k^\beta - \nabla v^\beta ) \mathrm {d}x \mathrm {d}t \nonumber \\&= \iint _{\Omega _T} \zeta A(v_k,\nabla v_k^\beta ) \cdot (\nabla v_k^\beta - \nabla v^\beta ) \mathrm {d}x \mathrm {d}t \nonumber \\&\quad - \iint _{\Omega _T} \zeta A(v,\nabla v^\beta ) \cdot (\nabla v_k^\beta - \nabla v^\beta )\mathrm {d}x \mathrm {d}t \nonumber \\&\quad + \iint _{\Omega _T} \zeta [A(v,\nabla v^\beta )-A(v_k,\nabla v^\beta )] \cdot (\nabla v_k^\beta - \nabla v^\beta ) \mathrm {d}x \mathrm {d}t \nonumber \\&=: \text{ I}_k + \text{ II}_k + \text{ III}_k, \end{aligned}$$

where $\nu = \max \{1,\frac{2}{p}\}$ and the constant *c* is independent of *k*. From Lemma 6.1, we know that the subsequence $(v_{k_j}^\beta )$ converges weakly in $L^p(0,T;W^{1,p}(\Omega ))$ to $v^\beta$ and that $v_{k_j}$ converges strongly in $L^{q}(\Omega _T)$ for any $q\ge 1$ and pointwise a.e. in $\Omega _T$ to *v*. Since $\zeta A(v,\nabla v^\beta )\in L^{p'}(\Omega _T;\mathbb {R}^n)$, the weak convergence $v_{k_j}^\beta \rightharpoondown v^\beta$ in $L^p(0,T;W^{1,p}(\Omega ))$ shows that the second term on the right-hand side of (6.3) vanishes in the limit $j\rightarrow \infty$. More precisely, we have

$$\begin{aligned} \lim _{j\rightarrow \infty } \text{ II}_{k_j} \equiv \lim _{j\rightarrow \infty } \iint _{\Omega _T} \zeta A(v,\nabla v^\beta ) \cdot (\nabla v_{k_j}^\beta - \nabla v^\beta )\mathrm {d}x \mathrm {d}t = 0. \end{aligned}$$

To treat the third term on the right-hand side of (6.3), we note that Lemma 5.6, the pointwise a.e. convergence $v_{k_j}\rightarrow v$ and the dominated convergence theorem guarantee that $A(v_{k_j},\nabla v^\beta )$ converges to $A(v,\nabla v^\beta )$ strongly in $L^{p'}$. The quantity $\nabla v_k^\beta -\nabla v^\beta$ stays bounded in $L^p$ due to Lemma 5.9, so by Hölder’s inequality we conclude that

$$\begin{aligned} \lim _{j\rightarrow \infty } \text{ III}_{k_j} \equiv \lim _{j\rightarrow \infty } \iint _{\Omega _T} \zeta \big [A(v,\nabla v^\beta )-A(v_{k_j},\nabla v^\beta )\big ] \cdot (\nabla v_{k_j}^\beta - \nabla v^\beta ) \mathrm {d}x \mathrm {d}t = 0. \end{aligned}$$

It only remains to control the first term on the right-hand side of (6.3). This term can be rewritten as

$$\begin{aligned} \text{ I}_{k}&= \iint _{\Omega _T} \zeta A(v_k,\nabla v_k^\beta ) \cdot \big (\nabla v_k^\beta - \nabla \llbracket v^\beta \rrbracket _h\big ) \mathrm {d}x \mathrm {d}t \\&\quad + \iint _{\Omega _T} \zeta A(v_k,\nabla v_k^\beta ) \cdot \big (\nabla \llbracket v^\beta \rrbracket _h-\nabla v^\beta \big ) \mathrm {d}x \mathrm {d}t \\&=: \text{ I}_{k}^{(1)} + \text{ I}_{k}^{(2)}, \end{aligned}$$

where $\llbracket v^\beta \rrbracket _h$ denotes the exponential time mollification defined in (3.4). Since $A(v_k,\nabla v_k^\beta )$ is bounded in $L^{p'}$ uniformly in *k*, we can use Hölder’s inequality to estimate the second term on the right-hand side as

$$\begin{aligned} |\text{ I}_{k}^{(2)}| \le \Vert A(v_k,\nabla v_k^\beta )\Vert _{L^{p'}(\Omega _T)} \Vert \nabla \llbracket v^\beta \rrbracket _h-\nabla v^\beta \Vert _{L^p(\Omega _T)} \le c\,\Vert \nabla \llbracket v^\beta \rrbracket _h-\nabla v^\beta \Vert _{L^p(\Omega _T)}, \end{aligned}$$

with a constant *c* independent of *k*. In order to treat the first term, we use the modified weak formulation (5.25) with the comparison function $w_{h,k}$ defined by $w_{h,k}^\beta =k^{-\beta }+\llbracket v^\beta \rrbracket _h$. The choice of comparison function requires some justification. We know that $k^{-\beta }+\llbracket v^\beta \rrbracket _h\in k^{-\beta }+L^p(0,T;W_0^{1,p}(\Omega ))$ since $v^\beta \in L^p(0,T;W_0^{1,p}(\Omega ))$. By Lemma 3.1 (iii), the exponential time mollification preserves this space. Moreover, by Lemma  3.1 (ii) and the boundedness of *v* we see that $\partial _t w_{h,k}^\beta \in L^\frac{\beta +1}{\beta }(\Omega _T)$. Thus, the comparison function $w_{h,k}$ is admissible, so that

$$\begin{aligned} \text{ I}_{k}^{(1)}&= \iint _{\Omega _T} \Big [\zeta ' \mathfrak {b}\big [v_k,w_{h,k}\big ] - \zeta \partial _t \llbracket v^\beta \rrbracket _h(v_k-w_{h,k}) \Big ]\mathrm {d}x\mathrm {d}t \\&\quad + \iint _{\Omega _T} \zeta \Big [ f\big (v_k^\beta -w_{h,k}^\beta \big ) + k^{-\alpha } |\nabla z|^{p-2}\nabla z \cdot \big (\nabla v_k^\beta -\nabla \llbracket v^\beta \rrbracket _h\big )\Big ] \mathrm {d}x\mathrm {d}t . \end{aligned}$$

By the convergence properties of $v_{k_j}$, we obtain

$$\begin{aligned} \limsup _{j\rightarrow \infty } \text{ I}_{k_j}^{(1)}&= \iint _{\Omega _T} \Big [\zeta ' \mathfrak {b}\big [v,(\llbracket v^\beta \rrbracket _h)^{\frac{1}{\beta }}\big ] - \zeta \partial _t \llbracket v^\beta \rrbracket _h\big (v-(\llbracket v^\beta \rrbracket _h)^{\frac{1}{\beta }}\big ) \Big ]\mathrm {d}x\mathrm {d}t \\&\quad + \iint _{\Omega _T} \zeta f \big (v^\beta -\llbracket v^\beta \rrbracket _h\big ) \mathrm {d}x \mathrm {d}t \\&\le \iint _{\Omega _T} \zeta ' \mathfrak {b}\big [v,(\llbracket v^\beta \rrbracket _h)^{\frac{1}{\beta }}\big ] \mathrm {d}x\mathrm {d}t + \iint _{\Omega _T} \zeta f \big (v^\beta -\llbracket v^\beta \rrbracket _h\big ) \mathrm {d}x \mathrm {d}t, \end{aligned}$$

where in the last line the term involving the time derivative has been omitted, since it is nonpositive by Lemma 3.1 (ii). Combining the last two estimates and joining the previously obtained bounds and convergence properties for $\text{ I}_{k_j}$ – $\text{ III}_{k_j}$ with (6.3), we end up with

$$\begin{aligned}&\limsup _{j\rightarrow \infty } \bigg [\iint _{\Omega _T} \zeta |\nabla v_{k_j}^\beta -\nabla v^\beta |^p\mathrm {d}x \mathrm {d}t\bigg ]^\nu \\&\quad \le c\big \Vert \nabla \llbracket v^\beta \rrbracket _h-\nabla v^\beta \big \Vert _{L^p(\Omega _T)}\\&\qquad + c\iint _{\Omega _T} \Big [\zeta ' \mathfrak {b}\big [v,(\llbracket v^\beta \rrbracket _h)^{\frac{1}{\beta }}\big ] + \zeta f \big (v^\beta -\llbracket v^\beta \rrbracket _h\big )\Big ] \mathrm {d}x \mathrm {d}t. \end{aligned}$$

By Lemma 3.1 (iii), we see that the right-hand side converges to zero as $h\rightarrow 0$. Thus, we have shown that

$$\begin{aligned} \lim _{j\rightarrow \infty }\iint _{\Omega _T} \zeta |\nabla v_{k_j}^\beta -\nabla v^\beta |^p\mathrm {d}x \mathrm {d}t = 0. \end{aligned}$$

This proves the claim of the lemma, since for any closed subinterval $I \subset (0,T)$ we can choose $\zeta \in C^\infty _0(0,T;[0,1])$ such that $\xi |_I=1$. In this case, $\chi _I\le \xi$ and the result follows. $\square$

### Lemma 6.3

The function *v* obtained in Lemma 6.1 satisfies

6.4 $$\begin{aligned} \iint _{\Omega _T} \big [A(v,\nabla v^\beta )\cdot \nabla \varphi - v\partial _t \varphi \big ]\mathrm {d}x\mathrm {d}t =\iint _{\Omega _T} f\varphi \mathrm {d}x\mathrm {d}t + \int _\Omega \Psi \varphi (0)\mathrm {d}x, \end{aligned}$$

for every $\varphi \in C^\infty (\bar{\Omega }\times [0,T])$ with support contained in $K\times [0,\tau ]$ where $K\subset \Omega$ is compact and $\tau \in (0,T)$.

### Proof

We fix a test function $\varphi$ as above, and note that it is sufficient to consider functions satisfying $|\nabla \varphi |\le 1$. Recall that $v_{k_j}$ satisfies (5.24) with $k=k_j$ and *j* sufficiently large. The goal is to pass to the limit $j\rightarrow \infty$. The limit

$$\begin{aligned} \lim _{j\rightarrow \infty } \iint _{\Omega _T} v_{k_j}\partial _t \varphi \mathrm {d}x \mathrm {d}t = \iint _{\Omega _T} v\partial _t \varphi \mathrm {d}x \mathrm {d}t \end{aligned}$$

follows from the $L^1$-convergence of $v_{k_j}$ to *v* and the limits

$$\begin{aligned}&\lim _{j\rightarrow \infty } k_j^{-\alpha }\iint _{\Omega _T} |\nabla z|^{p-2}\nabla z\cdot \nabla \varphi \mathrm {d}x \mathrm {d}t = 0,\\&\lim _{j\rightarrow \infty }\int _\Omega v_{k_j}(0)\varphi (0)\mathrm {d}x = \int _\Omega \Psi \varphi (0)\mathrm {d}x, \end{aligned}$$

are trivial (recall that $v_k(0)=\Psi +\tfrac{1}{k}$). It remains to treat the elliptic term. For this, we abbreviate

$$\begin{aligned} \mathcal {A}_k := A(v_k,\nabla v_k^\beta ) - A(v,\nabla v^\beta ) \end{aligned}$$

and note that for any $\delta \in (0,\tau )$ we can estimate

6.5 $$\begin{aligned} \iint _{\Omega _T}&|A(v_k,\nabla v_k^\beta )\cdot \nabla \varphi - A(v,\nabla v^\beta )\cdot \nabla \varphi |\mathrm {d}x \mathrm {d}t\\ \nonumber&\le \iint _{\Omega \times [0,\delta ]} |\mathcal {A}_k|\mathrm {d}x \mathrm {d}t + \iint _{\Omega \times [\delta ,\tau ]} |\mathcal {A}_k|\mathrm {d}x \mathrm {d}t, \end{aligned}$$

where we have used the Cauchy–Schwarz inequality and the bound on $\nabla \varphi$. Let $\varepsilon >0$. Due to the definition of *A*, and the uniform bounds obtained in Lemma 5.6 and Lemma 5.9, the integrand in the first term on the right-hand side of (6.5) is bounded in the $L^{p'}$-norm independently of *k*, so Hölder’s inequality allows us to conclude that for sufficiently small $\delta >0$, the bound

6.6 $$\begin{aligned} \iint _{\Omega \times [0,\delta ]} |\mathcal {A}_k|\mathrm {d}x \mathrm {d}t<\frac{\varepsilon }{2}, \end{aligned}$$

is satisfied independently of $k\in \mathbb {N}$. With such a fixed $\delta$, we now estimate the second term on the right-hand side of (6.5) as

6.7 $$\begin{aligned}&\iint _{\Omega \times [\delta ,\tau ]} |\mathcal {A}_k|\mathrm {d}x \mathrm {d}t \nonumber \\&\quad \le \iint _{\Omega \times [\delta ,\tau ]} |A(v_k,\nabla v_k^\beta ) - A(v_k,\nabla v^\beta )|\mathrm {d}x \mathrm {d}t\nonumber \\&\qquad + \iint _{\Omega \times [\delta ,\tau ]} |A(v_k,\nabla v^\beta ) - A(v,\nabla v^\beta )|\mathrm {d}x \mathrm {d}t. \end{aligned}$$

We will use Lemma 3.6 to treat both terms. This lemma shows that the integrand in the first term may be estimated as

$$\begin{aligned}&|A(v_k,\nabla v_k^\beta ) - A(v_k,\nabla v^\beta )|\\&\quad \le c \big (|\nabla v^\beta + \beta v_k^{\beta -1}\nabla z| + |\nabla v^\beta -\nabla v_k^\beta |\big )^{p-2}|\nabla v^\beta -\nabla v_k^\beta | \\&\quad \le c|\nabla v^\beta -\nabla v_k^\beta |^{p-1}+c\,b_p|\nabla v^\beta + \beta v_k^{\beta -1}\nabla z|^{p-2}|\nabla v^\beta -\nabla v_k^\beta |, \end{aligned}$$

where $c=c(p)$ and $b_p=0$ if $p<2$ and $b_p=1$ if $p\ge 2$. Convergence of the integral of the first term is clear as $k=k_j\rightarrow \infty$ due to Lemma 6.2. In the case $p\ge 2$, the integral of the second term is treated using Lemmas 5.6, 6.2 and Hölder’s inequality:

$$\begin{aligned}&\iint _{\Omega \times [\delta ,\tau ]} |\nabla v^\beta + \beta v_k^{\beta -1}\nabla z|^{p-2}|\nabla v^\beta -\nabla v_k^\beta | \mathrm {d}x\mathrm {d}t \\&\quad \le \bigg [\iint _{\Omega \times [\delta ,\tau ]}(|\nabla v^\beta |+\beta L^{\beta -1}|\nabla z |)^\frac{p(p-2)}{p-1} \mathrm {d}x \mathrm {d}t\bigg ]^\frac{p-1}{p} \Vert \nabla v^\beta -\nabla v_k^\beta \Vert _{L^p(\Omega \times [\delta ,\tau ])}. \end{aligned}$$

The exponent of the integral is less than *p*, so the integral is a finite number. These considerations show that the first term of (6.7) converges to zero as $k\in \{k_j\,|\,j\in \mathbb {N}\}$ approaches infinity. The integrand in the second term of (6.7) can be estimated using Lemma 3.6 as

$$\begin{aligned} |A(v_k,\nabla v^\beta ) - A(v,\nabla v^\beta )|&\le c\big (|\nabla v^\beta +\beta v^{\beta -1}\nabla z| \\&\quad + |\nabla z||v^{\beta -1}-v_k^{\beta -1}|\big )^{p-2}|\nabla z||v^{\beta -1}-v_k^{\beta -1}| \\&\le c |\nabla z|^{p-1} |v^{\beta -1}-v_k^{\beta -1}|^{p-1} \\&\quad + c\,b_p |\nabla v^\beta +\beta v^{\beta -1}\nabla z|^{p-2}|\nabla z||v^{\beta -1}-v_k^{\beta -1}|, \end{aligned}$$

where $c=c(p)$. The terms on the right-hand side can be treated by the dominated convergence theorem. Hence, for $k=k_j$ and for all sufficiently large *j*, we have

6.8 $$\begin{aligned} \iint _{\Omega \times [\delta ,\tau ]} |\mathcal {A}_k|\mathrm {d}x \mathrm {d}t < \frac{\varepsilon }{2}. \end{aligned}$$

Taking into account (6.5), (6.6) and (6.8), we have shown that

$$\begin{aligned} \lim _{j\rightarrow \infty } \iint _{\Omega _T}|A(v_{k_j},\nabla v_{k_j}^\beta )\cdot \nabla \varphi - A(v,\nabla v^\beta )\cdot \nabla \varphi |\mathrm {d}x \mathrm {d}t = 0. \end{aligned}$$

Taking into account all these limits, we have confirmed (6.4). $\square$

Now, we are ready to prove the main theorem.

### Proof of Theorem 2.2

We will show that the function *v* obtained in Lemma 6.1 is a weak solution to the Cauchy–Dirichlet problem (2.6) in the sense of Definition 2.1. Lemma 6.3 shows that (2.7) is valid, since for test functions $\varphi \in C^\infty _0(\Omega _T)$ the second term on the right-hand side of (6.4) vanishes. Moreover, by Lemma 3.11, we know that $v\in C([0,T];L^{\beta +1}(\Omega ))$. It only remains to show that $v(0)=\Psi$. Using (6.4) with the test function $\varphi (x)\zeta _\varepsilon (t)$ where $\varphi \in C^\infty _0(\Omega )$ and

$$\begin{aligned} \zeta _\varepsilon (t)={\left\{ \begin{array}{ll} \frac{1}{\varepsilon }(\varepsilon -t), &{}t\in [0,\varepsilon ], \\ 0, &{}t\ge \varepsilon , \end{array}\right. } \end{aligned}$$

one obtains

$$\begin{aligned} \lim _{\varepsilon \rightarrow 0} \frac{1}{\varepsilon }\int ^\varepsilon _0\int _\Omega v(x,t)\varphi (x)\mathrm {d}x\mathrm {d}t = \int _\Omega \Psi \varphi \mathrm {d}x. \end{aligned}$$

On the other hand, since $v\in C([0,T];L^{\beta +1}(\Omega ))$, we see that also

$$\begin{aligned} \lim _{\varepsilon \rightarrow 0} \frac{1}{\varepsilon }\int ^\varepsilon _0\int _\Omega v(x,t)\varphi (x)\mathrm {d}x\mathrm {d}t = \int _\Omega v(0)\varphi \mathrm {d}x \end{aligned}$$

holds. Since $\varphi \in C^\infty _0(\Omega )$ is arbitrary, it follows that $v(0)=\Psi$.
